# Activin A Is Essential for Neurogenesis Following Neurodegeneration

**DOI:** 10.1002/stem.80

**Published:** 2009-06

**Authors:** Andrea Abdipranoto-Cowley, Jin Sung Park, David Croucher, James Daniel, Susan Henshall, Sally Galbraith, Kyle Mervin, Bryce Vissel

**Affiliations:** a Neuroscience Program, The Garvan Institute of Medical ResearchSydney, Australia; b Faculty of Medicine, University of New South WalesSydney, Australia; c Cancer Program, The Garvan Institute of Medical ResearchSydney, Australia; d School of Mathematics and Statistics, University of New South WalesSydney, Australia

**Keywords:** Activin A, Anti-inflammatory, Astrocytes, Bone morphogenic protein, BMP receptors, Follistatin, Gliosis, Microglia, Neurodegeneration, Neurogenesis, Regeneration, Stem cells, Transforming growth factor-β, Inflammation, Neuroinflammation

## Abstract

It has long been proposed that excitotoxicity contributes to nerve cell death in neurodegenerative diseases. Activin A, a member of the transforming growth factor-β superfamily, is expressed by neurons following excitotoxicity. We show for the first time that this activin A expression is essential for neurogenesis to proceed following neurodegeneration. We found that intraventricular infusion of activin A increased the number of newborn neurons in the dentate gyrus, CA3, and CA1 layers of the normal adult hippocampus and also, following lipopolysaccharide administration, had a potent inhibitory effect on gliosis in vivo and on microglial proliferation in vivo and in vitro. Consistent with the role of activin A in regulating central nervous system inflammation and neurogenesis, intraventricular infusion of follistatin, an activin A antagonist, profoundly impaired neurogenesis and increased the number of microglia and reactive astrocytes following onset of kainic acid-induced neurodegeneration. These results show that inhibiting endogenous activin A is permissive for a potent underlying inflammatory response to neurodegeneration. We demonstrate that the anti-inflammatory actions of activin A account for its neurogenic effects following neurodegeneration because co-administration of nonsteroidal anti-inflammatory drugs reversed follistatin's inhibitory effects on neurogenesis in vivo. Our work indicates that activin A, perhaps working in conjunction with other transforming growth factor-β superfamily molecules, is essential for neurogenesis in the adult central nervous system following excitotoxic neurodegeneration and suggests that neurons can regulate regeneration by suppressing the inflammatory response, a finding with implications for understanding and treating acute and chronic neurodegenerative diseases.

## INTRODUCTION

Neurogenesis persists in distinct regions of the adult brain and is regulated by experience-dependent processes including learning, exercise, environmental enrichment, and stress [1, 2]. Emerging evidence suggests that neurogenesis may be impaired in neurodegenerative disorders such as Parkinson's disease and Alzheimer's disease and this might contribute to the pathogenesis of these chronic neurodegenerative disorders [3]. Meanwhile, acute injury to the central nervous system (CNS), such as that which occurs following a stroke, is followed by enhanced neural progenitor cell proliferation and neurogenesis. It has been speculated that this neurogenesis may contribute to the recovery that is observed [3]. It is unclear why neurogenesis proceeds after acute neurodegeneration but is impaired in chronic disease states. Although there is much interest in the potential for stem and progenitor cell-based therapy of the human CNS, progress requires that we must first understand the mechanisms that regulate neurogenesis in neurodegenerative disease conditions. Given its potential importance in brain function, disease, and therapy, elucidating the molecular mechanisms that regulate neurogenesis is imperative.

Excitotoxicity is believed to contribute to cell loss in neurodegenerative disorders [4]. Kainic acid (KA)-induced neurodegeneration in the hippocampus provides a powerful model for investigating the molecular mechanisms of excitotoxicity-induced neurodegeneration and neurogenesis. Systemic or intracerebral injections of KA result in a well-characterized pattern of neuronal cell loss in the hippocampus [5, 6], which is followed by extensive cell proliferation and neurogenesis in the dentate gyrus (DG) and hippocampal CA1 pyramidal cell layer [7–10]. Although the functional significance of neurogenesis observed in the KA model is still to be elucidated [1], the model allows investigation of the molecular mechanisms that regulate proliferation of neural progenitors in neurodegenerative disorders.

Very little is known of the mechanisms that regulate neurogenesis in the adult in neurodegenerative conditions. In this study we provide evidence for the critical role of activin A in regulating neurogenesis after KA-induced neurodegeneration. Activin A, a member of the transforming growth factor-β (TGFβ) superfamily, is known to regulate neural cell proliferation and differentiation during embryogenesis. A number of studies have shown that activin A expression is increased in principal neurons in the hippocampus of adult rodents in models of transient cerebral ischemia and hypoxia and also after KA treatment [11–14]. Previous in vitro studies have suggested that activin A stimulates the formation of new astrocytes [15]. Here, however, we provide evidence in support of an important but indirect role of activin A in neurogenesis following KA-induced neurodegeneration in the adult hippocampus in vivo. Our data suggest that activin A has an anti-inflammatory role and that this, possibly in concert with the action of other TGFβ superfamily molecules, is essential for neurogenesis following excitotoxic neurodegeneration. Since activin A is expressed by neurons [11–14], ours is the first study demonstrating a neural response that promotes neurogenesis through a potent anti-inflammatory action in vivo.

## MATERIALS AND METHODS

### Animals

Male C57Bl/6 mice aged 8-16 weeks were obtained from the Animal Resources Centre in Western Australia. All animal experiments were performed with the approval of the Garvan Institute and St. Vincent's Hospital Animal Ethics Committee, in accordance with National Health and Medical Research Council animal experimentation guidelines and the Australian Code of Practice for the Care and Use of Animals for Scientific Purposes (2004).

### Intracerebroventricular Injection of Kainic Acid and Lipopolysaccharide

Animals were anesthetized with ketamine (8.7 mg/ml; Mavlab, Slacks Creek, QLD, http://www.mavlab.com.au) and xylazine (2 mg/ml; Troy Laboratories Pty Ltd, Smithfield, Australia, http://www.troylab.com.au). Mice received a single unilateral stereotaxic injection of KA (0.2 μg, 1 μg/μl in phosphate buffered saline (PBS), pH 7.4; Ocean Produce International, Nova Scotia, CA, http://www.oceanproduce.com), lipopolysaccharide (LPS, 5 μg in PBS; Sigma-Aldrich, St. Louis, http://www.sigmaaldrich. com), or vehicle control (PBS) into the right lateral ventricle at Antero-Posterior (AP) −2.0 mm, Medio-Lateral (ML) −2.9 mm, Dorso-Ventral (DV) −3.8 mm (supporting information Fig. S1a). Animals that received KA only experienced seizure-like symptoms postsurgery ranging from tail rigidity to circling for 2–4 hours.

### Osmotic Micropump Implantation

Forty-eight hours after the injection of KA, osmotic micropumps (ALZET, Cupertino, CA, http://www.alzet.com) filled with activin A (12.25 ng/μl; R&D Systems Inc., Minneapolis, http://www.rndsystems.com), follistatin-288 (FS-288, 18.2 ng/μl; R&D Systems Inc.), or vehicle control (0.1% bovine serum albumin (BSA) in PBS) were implanted subcutaneously along the back of the neck. An infusion cannula (PlasticsOne, Roanoke, UA, http://www.plastics1.com) connected to the micropump was placed in the right lateral ventricle at AP −0.26 mm, ML −1.0 mm (supporting information Fig. S1a). The pump was removed 72 hours later and the animals were sutured and single-housed until they were sacrificed.

### Administration of Bromodeoxyuridine

We used the protocol developed by Cameron and McKay [16]. Bromodeoxyuridine (BrdU; Sigma-Aldrich) was administered as an i.p. injection of 300 mg/kg (0.9% saline) every 8 hours for 3 days beginning the morning after osmotic micropump implantation.

### Administration of Nonsteroidal Anti-inflammatory Drugs

Prior to implantation of osmotic micropumps containing recombinant mouse FS-288, animals received an i.p. injection of indomethacin (1 mg/kg; Sigma-Aldrich) and minocycline (50 mg/kg; Sigma-Aldrich) in 10% dimethyl sulfoxide. A single minocycline slow release pellet (75 mg; Innovative Research of America, Sarasota, FL, http://www.innovrsrch.com) was implanted subcutaneously concurrent to pump implantation. Animals received a second injection of indomethacin (1 mg/kg) and minocycline (25 mg/kg) following pump implantation. During FS-288 administration, animals received 12 hourly i.p. injections of indomethacin (1 mg/kg) and minocycline (25 mg/kg). After removal of (FS-288)-containing osmotic micropumps, animals received 12 hourly i.p. injections of minocycline only (25 mg/kg) for the next 2 days, whereas the pellet remained implanted for the entire 42 days.

### Tissue Preparation

Mice were anesthetized and perfused transcardially with ice-cold saline and then with 4% paraformaldehyde (4% PFA). Brains were harvested, postfixed (4% PFA) for 4 hours, and cryoprotected (30% sucrose). Tissue was cryosectioned at 40 μm and stored in PBS with 0.02% sodium azide at 4°C.

### Antibodies

The following antibodies were used. Mouse antibodies: anti-NeuN (NeuN, neuronal nuclei; Chemicon, Temecula, CA, http://www.chemicon.com). Rat antibodies: anti-BrdU (Abcam, Cambridge, U.K., http://abcam.com) and anti-CD11β (AbD Serotec Ltd., Oxford, U.K., http://www.abdserotec.com). Rabbit antibodies: anti-S100β (DAKO, Glostrup, Denmark, http://www.dako.com) and anti-GFAP (GFAP, glial fibrillary acidic protein; DAKO). Goat antibodies: anti-ActRIIb (ActRIIb, activin receptor IIb; Santa Cruz Biotechnology Inc., Santa Cruz, CA, http://www.scbt.com) and anti-Alk2 (Alk2, activin-like kinase receptor 2; Santa Cruz Biotechnology Inc.). Secondary antibodies were obtained from Invitrogen (Carlsbad, CA, http://www.invitrogen. com).

### Immunofluorescence

For DNA denaturation tissue sections were pretreated with TACs nuclease (1 hour, 37°C; R&D Systems Inc.) and neutralized with PBS. Sections were blocked in 3% BSA + 0.25% Triton X-100 in PBS [1 hour, room temperature (rt)]. Primary antibodies were applied in blocking solution for 3 days at 4°C. Antibodies were detected with Alexa Fluor tagged secondary antibodies. Tissue was counterstained using 4′,6-diamidino-2-phenylindole (DAPI; Invitrogen).

### Immunoperoxidase

Free-floating sections were incubated in 50% ethanol (20 minutes, rt) and then in 3% H_2_O_2_ (20 minutes, 4°C) prior to blocking in 3% BSA + 0.25% Triton X-100 in PBS. Sections were incubated with mouse anti-NeuN. Neuronal labeling was detected using biotinylated goat anti-mouse antibody and avidin-biotin peroxidase complex, followed by peroxidase detection (diaminobenzidine; Vector Laboratories, Burlingame, CA, http://www.vectorlabs.com).

### Stereology

For all population estimates we restricted our counts to analysis of the dorsal hippocampus at the positions between −1.34 mm and −2.3 from bregma. The regions sampled included the DG, CA3, CA1, and posterior periventricular area (pPV). The DG was defined as the area containing the granular cell layer and the hilus. The pPV region was defined as the area between the lateral ventricle and the CA3 and CA1 regions. All stereological cell counts were performed blind (a detailed discussion of the theory, approaches, considerations, and assumptions is outlined in the supporting information Materials and Methods section).

### Isolation of Mouse Primary Astrocytes and Microglia

Astrocytes and microglia were harvested from mixed glial cells following the procedure of Butovsky et al. as described previously [17]. For a detailed description of primary cell culture techniques, see supporting information Materials and Methods section.

### Stimulation of Astrocytes and Microglia

All growth factors were dissolved in 0.1% BSA in Dulbecco's PBS (D-PBS). Cells were treated with control solution, 10 μg/ml LPS, 100 ng/ml activin A, 1 μg/ml activin A, or a combination of 100 ng/ml activin A or 1 μg/ml activin A with 10 μg/ml LPS. Astrocytes were treated under serum (10% fetal bovine serum)-containing conditions for 48 hours when nitric oxide was measured using Griess reagent. Microglial stimulation was performed under serum-free conditions for 24 hours. Following treatment, microglia were fixed for immunocytochemistry analysis.

### Immunofluorescence Analysis

After treatment with the growth factors and/or LPS, cells were washed free of growth factor-/LPS-containing media with PBS. Glial cells were fixed by 4% PFA/4% sucrose (PBS, pH 7.4, 37°C, 8 minutes), permeabilized using 0.25% Triton X-100 for 5 minutes, and blocked using 10% BSA in PBS for 30 minutes. All primary antibodies were diluted in chilled 3% BSA in PBS and applied overnight at 4°C. Primary antibodies were detected using Alexa Fluor tagged secondary antibodies. Cells were counterstained with DAPI.

### Measurement of Cytokine Release

After 24 hours of treatment with the growth factors and/or LPS, media were harvested. Cytokine measurements were performed using the BD Cytometric Bead Array Mouse Inflammation Kit (BD Biosciences, San Diego, http://www.bdbiosciences.com) and FACsCanto according to manufacturers' protocols.

### Image Acquisition

All images were obtained using Leica DM IRE2 TCS SP2 AOBS inverted laser scanning confocal (Leica, Heerbrugg, Switzerland, http://www.leica.com). Images were compiled and analyzed using Adobe Photoshop CS2 (Adobe, San Jose, CA, http://www.adobe.com).

### Statistical Analysis

All statistical analysis was performed using the statistical package SPSS v11 (Graduate pack) (SPSS Inc., Chicago, IL, http://www.spss.com). All data sets were tested for normality using the Shapiro-Wilk test. Unless otherwise stated, pairwise comparisons were statistically analyzed using independent two-sample *t* tests with Bonferroni correction. The reason for our approach was that we were not interested in all pairwise comparisons, only the comparisons of treated groups with control. Hence, we only adjusted for the comparisons that were performed, not all possible pairwise comparisons. The Bonferroni procedure is a general method that can be applied in this situation. Used in this way, the Bonferroni procedure controls the type I error rate for each collection of pairwise comparisons that were actually performed [18].

## RESULTS

### Follistatin Inhibits Neurogenesis in the Intact and Injured Hippocampus

Kainic acid (KA) is an excitotoxin (supporting information Fig. S1) and neurogenesis is increased after KA-induced neurodegeneration in the hippocampal DG and in the hippocampal CA1 and CA3 neuronal layers (supporting information Fig. S2). We were interested in determining whether TGFβ superfamily molecules might play a role in regulating neurogenesis during the period after cell death has proceeded. As a first step, we investigated whether the expression of specific mRNAs encoding members of the TGFβ superfamily was increased 54 hours after KA injections (supporting information Table 1), when neurodegeneration had already proceeded. Of all mRNAs we measured, mRNAs encoding activin A showed the greatest increase in expression levels at this time point. The mRNA encoding the βA subunit that makes up activin A increased almost 24-fold in KA-treated hippocampi compared to that in hippocampi that received a control injection, although there were essentially no changes in the expression of mRNAs encoding the βB or α subunits that make up inhibin or activin B (supporting information Table 1). We therefore hypothesized that activin A may play a role in mediating the neurogenesis observed after KA-induced neurodegeneration.

**Figure 1:**
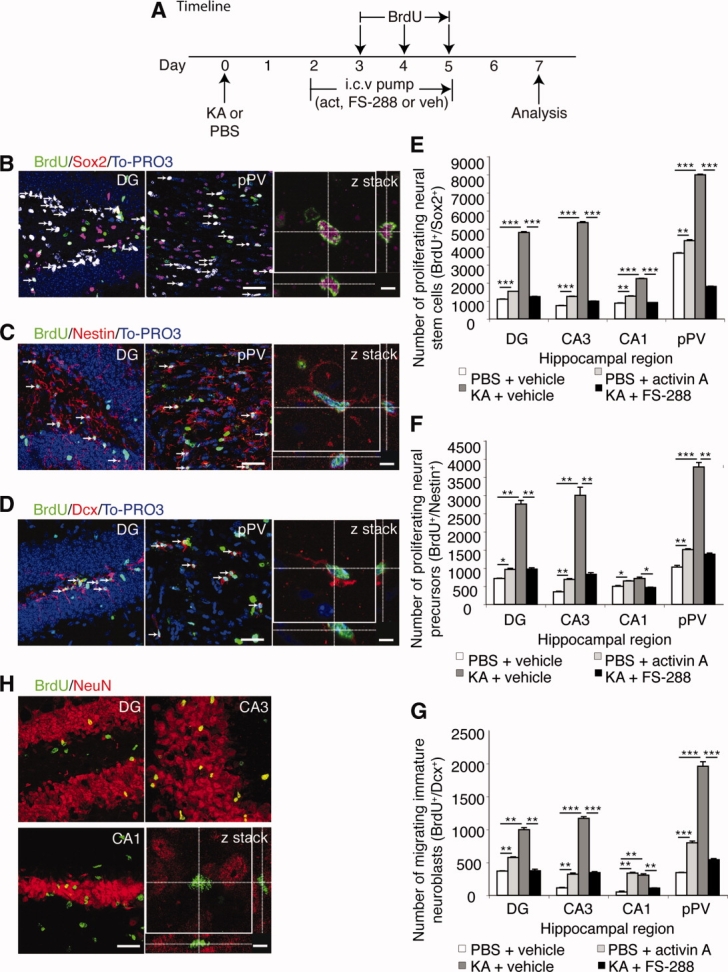
Activin A induces neural stem cell and precursor proliferation in the adult injured and intact hippocampus. **(A):** Experimental timeline: On day 0 animals received a single i.c.v. injection of KA or PBS control. Starting on day 2, an i.c.v. infusion of activin A, FS-288, or vehicle began and was continued for 3 days via an osmotic micropump. Animals received three times daily i.p. injections of BrdU beginning at 7 a.m. for the 3 days indicated. On day 7 the tissue was harvested for analysis. **(B**–**D):** Images of **(B)** proliferating multipotential neural stem cells co-expressing BrdU (green) and Sox2 (red), **(C)** proliferating neural precursor cells co-expressing BrdU (green) and nestin (red), and **(D)** proliferating immature migrating neuroblasts co-expressing BrdU (green) and doublecortin (red). For all confocal images, low-power scale bar = 50 μm and high-power scale bar = 5 μm. **(E–G):** Quantification of proliferating cells revealed that compared to their respective vehicle-treated controls [KA injected with vehicle (dark gray bars, *n* = 5); PBS injected with vehicle (white bars, *n* = 5)], infusion of FS-288 in KA-injected animals (*n* = 5, black bars) inhibited proliferation, whereas infusion of activin A in PBS-injected animals (*n* = 5, light gray bars) increased proliferation of **(E)** multipotential neural stem cells, **(F)** neural precursors, and **(G)** immature migrating neuroblasts in the DG, CA3, CA1, and pPV area. **(H):** Seven days after KA low- and high-power (confocal z stack) images show that cells did not co-express NeuN (red) and BrdU (green). Values for all graphs are mean ± SEM. ∗, *p* < .025; ∗∗, *p* < .005; ∗∗∗, *p* < .0005 (independent two-sample *t* test with Bonferroni correction). Abbreviations: BrdU, bromodeoxyuridine; Dcx, doublecortin; DG, dentate gyrus; FS-288, follistatin-288; i.c.v., intracerebroventricular; KA, kainic acid; NeuN, neuronal nuclei; PBS, phosphate buffered saline; pPV, posterior periventricular area.

Activin A is expressed by principal neurons and has neuroprotective effects following excitotoxicity [12, 13, 19]. Follistatin-288 (FS-288) is a high-affinity antagonist of activin A [20]. To investigate the effects of activin A on neurogenesis, we developed a protocol to study neurogenesis in which we first allowed neurodegeneration to be fully committed before administering activin A or FS-288 (Fig. 1A). In our protocol, a single dose of KA or PBS control was stereotaxically injected into the right lateral ventricle. The animals were then allowed to recover for 48 hours, during which time a reproducible cell loss occurred in the CA3 and CA1 pyramidal cell layers of the hippocampus (supporting information Fig. S1). Subsequently, vehicle, activin A, or FS-288 was infused into the lateral ventricle for 72 hours, during which time animals also received bromodeoxyuridine (BrdU) for 3 days to label dividing cells. We then collected tissue 7 days later for analysis.

The proliferative zones of the hippocampus contain the following: Sox2-expressing cells, considered to be authentic neural stem cells (NSCs) with multipotent and self-renewing NSC properties [21]; nestin-expressing cells, considered to be neural precursor cells (NPCs) [22]; and doublecortin (Dcx)-expressing cells, considered to be immature migrating neuroblasts [23]. These neural stem/precursor cells are observed in the subgranular zone (SGZ) of the DG and in the posterior periventricular area (pPV) and they have been shown to be capable of repopulating the DG and CA1 neuronal layers [7, 24]. Therefore, to assess the extent of neural stem/precursor proliferation after KA-mediated degeneration, we performed population estimates of cells labeled for BrdU and either Sox2, nestin, Dcx, or NeuN, using confocal microscopy and stereological techniques (see supporting information Materials and Methods section). As expected [7], we observed that KA-induced neurodegeneration was followed by a 337%, 120%, 634%, and 157% increase in the average number of proliferating multipotent NSCs (Fig. 1B, 1E), a 287%, 278%, 770%, and 44% increase in the average number of proliferating NPCs (Fig. 1C, 1F), and a 170%, 468%, 935%, and 479% increase in the average number of migrating immature neuroblasts (Fig. 1D, 1G), in the DG (including the SGZ) and pPV, and in the CA3 and CA1 neuronal layers, respectively, compared with animals that received no KA.

**Figure 2:**
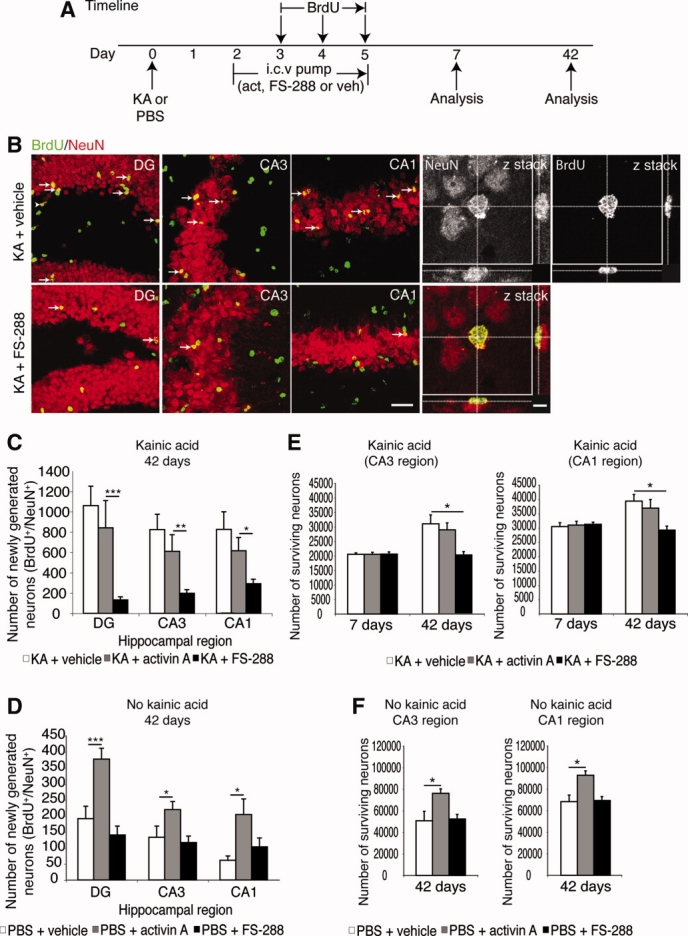
Activin A regulates long-term neurogenesis in the adult injured and intact hippocampus. **(A):** Experimental timeline (for details refer to Fig. 1). Tissue was analyzed at 7 days or 42 days. **(B):** Forty-two days after KA injection newborn neurons co-express the neuronal marker, NeuN (red), and the proliferative marker, BrdU (green), although less newborn neurons were seen in KA-treated animals that received FS-288 compared to animals that did not receive FS-288. Low-power scale bar = 50 μm and high-power scale bar = 5 μm. **(C):** Quantification revealed that infusion of FS-288 in KA-injected animals (*n* = 10, black bars) inhibited neurogenesis in the DG, CA3, and CA1, whereas infusion of activin A in KA-injected animals (*n* = 10, gray bars) had no effect on the number of new neurons compared to KA-injected controls (*n* = 11, white bars). **(D):** There was, however, increased neurogenesis in the DG, CA3, and CA1 regions of the dorsal intact hippocampus in activin A-treated animals (*n* = 10, gray bars), whereas (FS-288)-treated animals had no effect on neurogenesis (*n* = 10, black bars) compared to controls (*n* = 10, white bars). **(E):** Administration of activin A or FS-288, 2 days after KA-induced injury, did not alter neuron survival when administered by our protocol since there were no differences in the total neuron population observed in the CA3 or CA1 regions 7 days after KA in animals that received infusions of activin A (*n* = 8, gray bars) or FS-288 (*n* = 8, black bars) compared to KA-injected controls (*n* = 8, white bars). However, FS-288 administration prevented the subsequent increase in total neuron population observed 42 days after the KA-induced neurodegeneration, suggesting endogenous activin A expression increases total neuron population after KA neurodegeneration. **(F):** Meanwhile, activin A infusion following PBS injection (*n* = 8, gray bars) increased the total neuron population 42 days later in the CA3 and CA1 compared to their respective vehicle-treated controls (*n* = 8, white bars). Infusion of FS-288 following PBS injection (*n* = 8, black bars) had no effect on the total neuron population. Values for all graphs are mean ± SEM. ∗, *p* < .025; ∗∗, *p* < .005; ∗∗∗, *p* < .0005 (independent two-sample *t* test with Bonferroni correction). Abbreviations: BrdU, bromodeoxyuridine; DG, dentate gyrus; FS-288, follistatin-288; KA, kainic acid; NeuN, neuronal nuclei; PBS, phosphate buffered saline.

No BrdU-labeled mature neurons were observed in the DG, CA3 or CA1 or neuronal layers of the hippocampus 7 days after the KA-induced neurodegeneration (Fig. 1H), although many BrdU-labeled neurons were observed at 42 days (Fig. 2). Thus, in our protocol, BrdU did not label mature neuronal cells during the period of BrdU administration. BrdU is therefore not labeling dying neurons in our protocol. We confirmed that BrdU labeling was specific to proliferating cells because intracerebroventricular (i.c.v.) infusion of the mitotic inhibitor cytosine β-D-arabinofuranoside hydrochloride, which is expected to block cell proliferation in the brain, also greatly reduced BrdU labeling of centrally derived cells, but not of peripherally derived cells (supporting information Fig. S3).

Consistent with the significant role of endogenous activin A in promoting proliferation after neurodegeneration, infusion of FS-288, an activin A antagonist, 48 hours after KA injection, led to a 74%, 82%, 59%, and 77% reduction in the average number of proliferating multipotential NSCs (Fig. 1B, 1E), a 65%, 72%, 35%, and 63% reduction in the average number of proliferating NPCs (Fig. 1C, 1F), and a 62%, 71%, 64%, and 72% reduction in the average number of migrating immature neuroblasts (Fig. 1D, 1G) in the DG (including the SGZ) and pPV and in the CA3 and CA1 neuronal layers, respectively, compared with animals that received KA but no FS-288. Since FS-288 is an antagonist of activin A, these results strongly implicated activin A in neural stem/precursor cell proliferation after KA-induced neurodegeneration.

We, therefore, asked whether activin A could stimulate neural stem/precursor cell proliferation in the absence of KA-induced neurodegeneration. We found that infusion of activin A, 2 days after an i.c.v. saline injection, led to a 39%, 70%, 45%, and 16% increase in the average number of proliferating multipotential NSCs (Fig. 1B, 1E), a 26%, 50%, 30%, and 47% increase in the average number of proliferating NPCs (Fig. 1C, 1F), and a 56%, 184%, 536%, and 132% increase in the average number of proliferating immature neuroblasts (Fig. 1D, 1G) in the DG (including the SGZ) and pPV and in the CA3 and CA1 neuronal layers, respectively, compared to animals that received no activin A. These results confirmed that activin A could stimulate neural stem/precursor cell proliferation and subsequent early differentiation into immature migrating neuroblasts.

The BrdU-labeled NSCs, NPCs, and migrating neuroblasts observed at 7 days after KA injection (Fig. 1) transformed into mature BrdU-labeled neurons by 42 days (supporting information Fig. S2). The number of newly generated mature BrdU/NeuN labeled neurons in the CA1 and CA3 regions at 42 days appeared to be less than the number of neural stem/progenitor cells observed in the CA1, CA3, and pPV (Figs. 1, 2). This suggests some of the neural stem/progenitor cells either differentiated into other cell types in addition to neurons or underwent cell death. Nevertheless, the number of BrdU/NeuN labeled cells at 42 days after KA was significantly increased, compared to animals that received no KA (supporting information Fig. S2).

Since FS-288 inhibits neural stem/progenitor cell proliferation after KA-induced neurodegeneration (Fig. 1), we predicted that it would impair neurogenesis. Indeed, we found that FS-288 reduced the average number of newly generated neurons 42 days after KA injection by 88%, 76%, and 65% in the DG, CA3, and CA1 regions, respectively, compared to animals that received KA but no FS-288 (Fig. 2B, 2C). Meanwhile, infusion of activin A, 48 hours after KA-induced neurodegeneration had no additional effect on the extent of neurogenesis at 42 days, suggesting that endogenous activin A was already exerting a maximal effect after KA-induced neurodegeneration.

We also investigated whether the increase in neural stem/precursor cell proliferation caused by activin A infusion in the intact brain at 7 days (Fig. 1) led to increased neurogenesis at 42 days (Fig. 2D). As expected, there was a 96%, 65%, and 234% increase in the average number of newly generated neurons in the DG, CA3, or CA1 regions of the hippocampus of animals that received activin A, compared to animals that received no activin A after a PBS injection. Meanwhile, infusion of FS-288 did not have any effect on neurogenesis compared to vehicle-treated PBS-injected animals (Fig. 2D). Thus, although exogenously administered activin A can stimulate neurogenesis, it is not essential for neurogenesis in the normal, uninjured brain.

Activin A has neuroprotective effects if administered prior to or in conjunction with excitotoxic insult [12]. Importantly, however, we noted that the i.c.v. infusion of activin A or FS-288 beginning 48 hours after KA-induced neurodegeneration did not significantly alter neuronal survival in the CA3 and CA1 regions 7 days after KA injection, compared to controls (Fig. 2E). This confirmed that although activin A may be neuroprotective when administered prior to, or in conjunction with, excitotoxic injury [12], neither activin A nor FS-288 exerted effects on neuronal survival when administered 2 days after neurodegeneration. This strongly suggests that neurodegeneration was fully committed by the time point where we administered FS-288 or activin A in our studies. Activin A is, however, expressed more than 48 hours after KA injury (supporting information Table 1) and, as shown in Figures 1 and 2, is required for neurogenesis after neurodegeneration occurs.

An important observation is that although cell loss is observed after KA (supporting information Fig. S1), subsequent neurogenesis actually partly restored the total neuron populations in the CA3 and CA1 regions. Indeed, as shown in Figure 2E, the total neuron populations in the CA3 and CA1 regions increased between 7 days and 42 days post-KA, an effect that was not observed when neurogenesis was blocked by FS-288 infusion. Furthermore, infusion of activin A alone increased the total neuron population in the CA3 and CA1 regions of the hippocampus 42 days later, even in the absence of previous cell loss (Fig. 2F). These results show that newly generated neurons can contribute to an increase in the total neuron population after KA-induced neurodegeneration, that this increase is blocked by FS-288, and that 3 days of activin A infusion alone is sufficient to drive an increase in neuron numbers in the absence of neurodegeneration.

### Activin A Regulates Gliosis in the Injured Adult Hippocampus In Vivo

**Figure 3:**
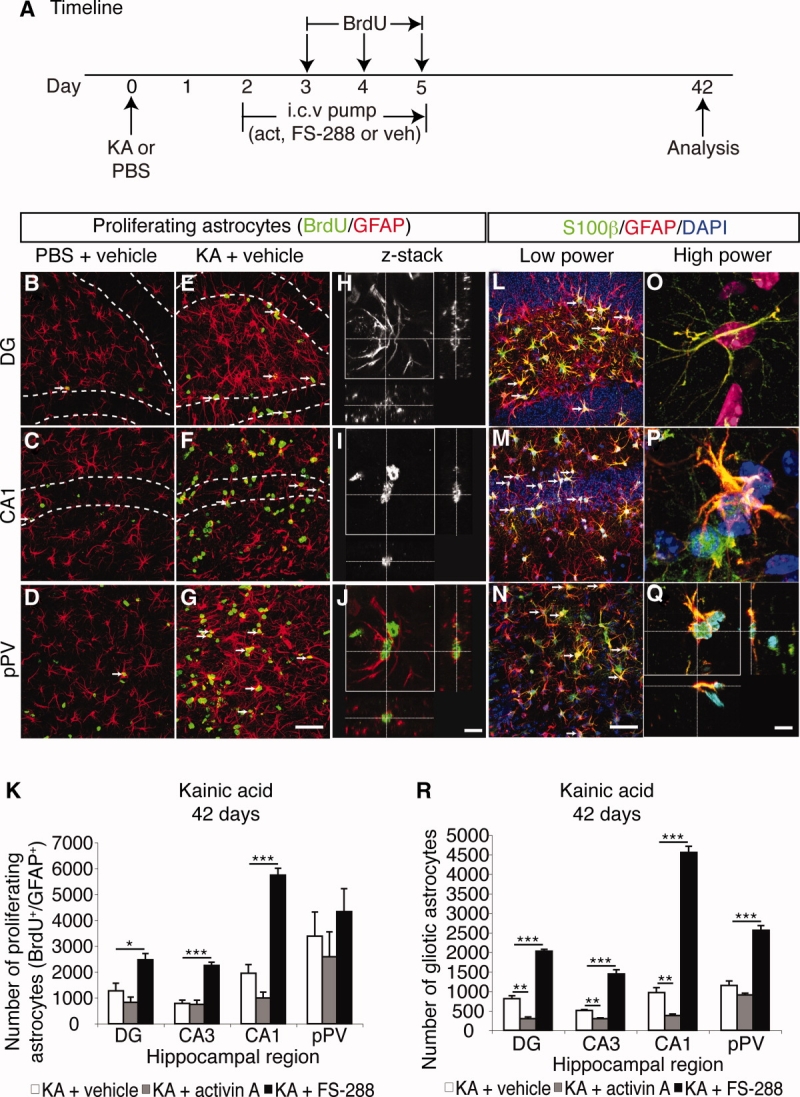
Activin A inhibits astrocyte proliferation in the injured hippocampus. **(A):** Experimental timeline. **(B–G):** Increased astrocyte proliferation (arrows) was observed in the DG, CA1 and pPV of animals that received KA compared to animals that did not receive KA. Scale bar = 50 μm. **(H–J):** Confocal z-stack images showed that proliferating astrocytes (**J**, overlay of **H** and **I**) co-expresss GFAP (red, **H**) and BrdU (green, **I**). Scale bar = 5 μm. **K**, Quantification revealed enhanced astrocyte proliferation in the DG, CA3, CA1 and pPV regions of the dorsal hippocampus in KA-injected animals that received FS-288 (*n* = 7, black bars) compared to KA-injected controls (*n* = 7, white bars), while, infusion of activin A following KA injection (*n* = 7, grey bars) had no significant effect on astrocyte proliferation compared to KA control animals. **L–Q**, Analysis of astrocytes with S100β and GFAP revealed extensive gliosis (arrows) in the DG, CA1 and pPV following KA-induced neurodegeneration. Scale bar = 50 μm. **(O–Q):** Images of astrocyte morphology show that **(O)** quiescent astrocytes possess long, thin processes while, **(P)** gliotic astrocytes displayed cellular hypertrophy and a shortening or thickening of processes and **(Q)** co-expressed BrdU (green), GFAP (red) and DAPI (blue). Scale bar = 5 μm. **R**, Quantification of astrocytes that co-expressed BrdU and GFAP and exhibited cellular hypertrophy revealed that infusion of FS-288 following KA injection (*n* = 7, black bars) enhanced gliosis, while infusion of activin A following KA injection (*n* = 7, grey bars) inhibited gliosis in the DG, CA3, CA1 and pPV regions of the dorsal hippocampus compared to KA-injected control animals (*n* =7, white bars). Values for all graphs as mean ± SEM. *: *p* < 0.025, **: *p* < 0.005, ***: *p* < 0.0005 (Independent two sample *t*-test with Bonferroni correction). Abbreviations: BrdU, bromodeoxyuridine; DAPI, 4′,6-diamidino-2-phenylindole; DG, dentate gyrus; FS-288, follistatin-288; GFAP, glial fibrillary acidic protein; KA, kainic acid; PBS, phosphate buffered saline; pPV, posterior periventricular area.

Injury to the adult CNS is accompanied by reactive gliosis, whereby astrocytes proliferate and undergo cellular hypertrophy (shortening and thickening of processes) [25]. To determine whether activin A had a role in this process, we first assessed whether activin A regulated the number of proliferating astrocytes in the DG, CA3, CA1, and pPV regions [7, 24]. We first analyzed changes in astrocyte proliferation (BrdU-positive/GFAP-positive) 42 days after the KA-induced neurodegeneration (Fig. 3A–3K). Consistent with the role of activin A in regulating astrocyte proliferation after KA-induced neurodegeneration, infusion of FS-288 following KA injection resulted in a 94%, 187%, and 194% increase in the average number of proliferating astrocytes in the DG, CA3, and CA1 regions, respectively, but not in the neurogenic pPV, when compared to KA-injected animals that received no FS-288 (Fig. 3K). Infusion of activin A itself had no additional effect on astrocyte proliferation in the hippocampal DG, CA3, CA1, and pPV regions after KA injection (Fig. 3K). The effect of activin A on astrocytes depends on there being some other inflammatory insult present since infusion of activin A did not affect the number of proliferating astrocytes in the hippocampus of animals that received no KA (data not shown).

The changes in astrocyte proliferation were associated with cellular hypertrophy that is characteristic of gliosis (Fig. 3L–3Q) since there were also changes in the number of proliferating astrocytes that exhibited gliotic morphology (shortened and thickened processes; Fig. 3P, 3Q) as opposed to a quiescent morphology (thin, elongated processes; Fig. 3O). Specifically, the average number of proliferating astrocytes with a gliotic morphology after KA-induced neurodegeneration was profoundly altered by infusion of FS-288, as this resulted in a 150%, 183%, 371%, and 123% increase in the average number of gliotic cells in the DG, CA3, CA1, and pPV regions, respectively, compared to vehicle-treated animals that received KA but no FS-288 (Fig. 3R). Infusion of activin A following KA-induced neurodegeneration resulted in a 63%, 42%, 61%, and 21% decrease in the average number of gliotic cells in the DG, CA3, CA1, and pPV regions, respectively, compared to vehicle-treated animals that received KA but no activin A (Fig. 3R). In the absence of KA-induced degeneration, activin A had no effect on the average number of proliferating astrocytes with gliotic morphology, as expected (data not shown).

### Activin A Regulates Microglial Numbers in the Hippocampus In Vivo

**Figure 4:**
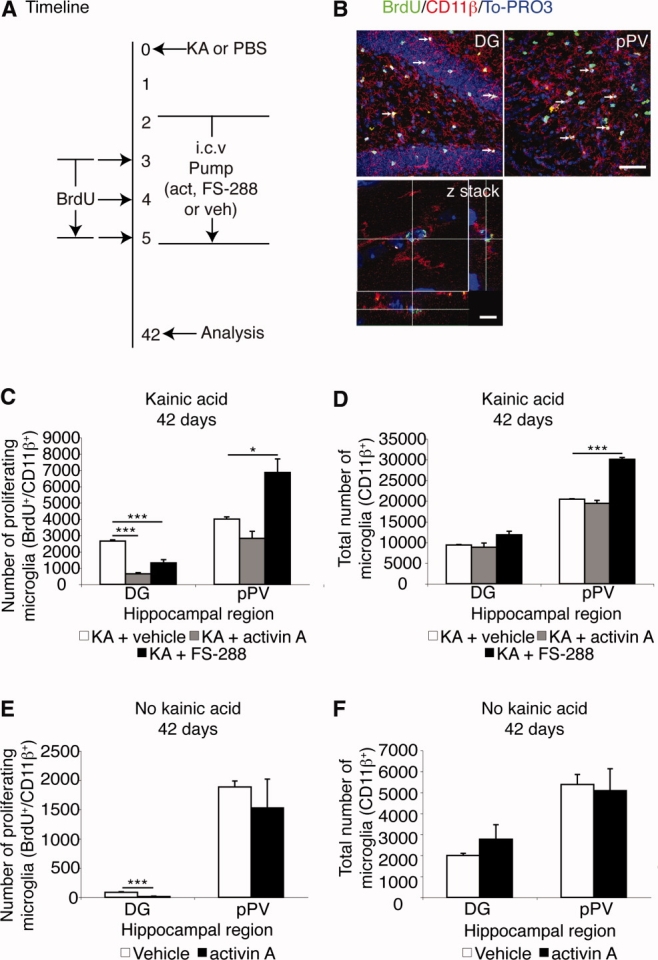
Activin A inhibits microglial proliferation in the intact and injured hippocampus. **(A):** Experimental timeline. **(B):** Proliferating microglia co-express the microglial marker, CD11β (red), and the proliferative cell marker, BrdU (green), counterstained with DAPI (blue). Low-power scale bar = 50 μm and high-power scale bar = 5 μm. **(C):** Quantification revealed that the number of proliferating microglia (BrdU^+^/CD11β^+^) over 3 days of BrdU treatment in the DG was inhibited by infusion of activin A (*n* = 5, gray bars) and FS-288 (*n* = 5, black bars), compared to controls (*n* = 5, white bars), whereas in the pPV, infusion of FS-288 in KA-injected animals stimulated microglial proliferation. **(D):** Infusion of activin A (*n* = 5, gray bars) or FS-288 (*n* = 5, black bars) in KA-injected animals did not change in the total microglial population in the DG compared to controls (*n* = 5, white bars), whereas in the pPV of KA-injected animals, FS-288 led to an increase in the total microglial population when compared to controls. **(E):** Infusion of activin A in animals that did not receive KA (*n* = 5, black bars) inhibited microglial proliferation over 3 days of BrdU in the DG but had no further effects on microglial proliferation in the pPV compared to controls (*n* = 7, white bars). **(F):** Infusion of activin A in animals that did not receive KA (*n* = 5, black bars) had no effect on the total microglial population in the DG and pPV despite decreased microglial proliferation in the DG compared to controls (*n* = 5, white bars). Values for all graphs are mean ± SEM. ∗, *p* < .025; ∗∗, *p* < .005; ∗∗∗, *p* < .0005 (independent two-sample *t* test with Bonferroni correction). Abbreviations: BrdU, bromodeoxyuridine; DAPI, 4′,6-diamidino-2-phenylindole; DG, dentate gyrus; FS-288, follistatin-288; KA, kainic acid; PBS, phosphate buffered saline; pPV, posterior periventricular area.

Gliosis is characteristic of central inflammation. Our finding that FS-288 increased gliosis and that activin A infusion reduced gliosis lead us to hypothesize that activin A may have anti-inflammatory actions. If true, then activin A would potentially regulate the microglial response to injury, which could in turn regulate gliosis. Thus, FS-288 infusion would be predicted to increase microglial numbers in the hippocampus. We, therefore, investigated the effects of activin A on microglial responses in the neurogenic regions, the DG and pPV, 42 days after the KA-induced neurodegeneration (Fig. 4A, 4B). In the pPV, we found that inhibition of activin A by FS-288 resulted in a 72% average increase of microglial proliferation compared to KA-injected animals that received no FS-288 (Fig. 4C) and this led to an expected 72% increase in the average total microglial population (Fig. 4D). In contrast, in the DG, there was an unexpected 50% decrease in average microglial proliferation (labeled over 3 days of BrdU), induced by FS-288 infusion, although the average total microglial population was not significantly changed (Fig. 4D). Therefore, microglial infiltration, or replication post-BrdU, must determine the number of microglia in the DG. Meanwhile, infusion of activin A after KA injection led to a 76% decrease in the average microglial proliferation in the DG (Fig. 4C) compared to that induced by KA alone, but this did not have a significant effect on the total number of microglial cells in the DG or pPV (Fig. 4D).

We also investigated the effect of activin A on microglial populations in animals that received no KA. Our data showed that infusion of activin A in the intact hippocampus had no significant effect on the total microglial population in either the DG or the pPV compared to vehicle-treated controls (Fig. 4F), although it did lead to an 86% average decrease in microglial proliferation in the DG (Fig. 4E). Collectively, our results indicate that activin A regulates microglial numbers in vivo, acting as an anti-inflammatory agent subsequent to the action of other inflammatory stimuli in the injured hippocampus.

### Activin A Exerts Anti-inflammatory Effects In Vivo

**Figure 5:**
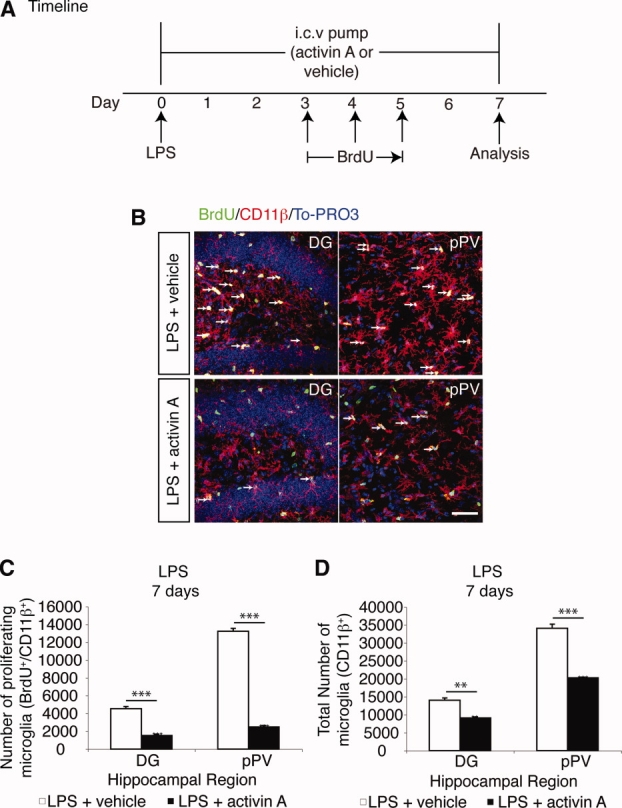
Activin A possesses anti-inflammatory properties in vivo. **(A):** Experimental timeline. On day 0 animals received a single i.c.v. injection of LPS. Also on day 0, a 7-day infusion of activin A or vehicle was initiated via osmotic micropump. During this period, animals received three times daily i.p. injections of BrdU for 3 days, beginning at 7:00 a.m. on day 3. On day 7 the tissue was harvested for immunohistochemical analysis. **(B):** Activin A infusion decreased the number of microglia (red) and the number of proliferating microglia (arrows, white) that co-labeled with BrdU (green), CD11β (red), and 4′,6-diamidino-2-phenylindole (blue), in the DG and pPV of LPS-injected animals that received activin A infusion, compared to LPS-injected animals that did not receive activin A. Scale bars = 50 μm. **(C, D):** Quantification supported observations that activin A in LPS-injected animals (*n* = 5, black bars) **(C)** inhibited microglial proliferation and **(D)** decreased the total microglial population in the DG and pPV compared to control animals (*n* = 5, white bars). Values for all graphs are mean ± SEM. ∗, *p* < .025; ∗∗, *p* < .005; ∗∗∗, *p* < .0005 (independent two-sample *t* test with Bonferroni correction). Abbreviations: BrdU, bromodeoxyuridine; DG, dentate gyrus; i.c.v., intracerebroventricular; KA, kainic acid; LPS, lipopolysaccharide; pPV, posterior periventricular area.

The toll-like receptor (TLR) pathway, in particular, TLR4 and TLR2, regulate microglial activation in vivo and in vitro [26]. Activation of this pathway has been implicated in inflammation in several neurodegenerative diseases and in stroke [27–29]. The injection of LPS into the CNS can be used to model these inflammatory effects because LPS activates TLR4 to induce microglial activation and the release of pro-inflammatory cytokines, stimulating inflammation in the hippocampus, without inducing any neuronal death [30]. To confirm that activin A has direct anti-inflammatory effects in vivo, we investigated whether activin A can suppress microglial activation by LPS. We infused activin A in a model of LPS inflammation following the protocol shown in Figure 5A. Our analysis confirmed that injection of LPS into the hippocampus, whether followed by infusion of vehicle or activin A, resulted in no significant loss of neurons in the DG, CA3, or CA1 regions of the hippocampus compared to PBS-injected vehicle-infused animals (supporting information Fig. S4). We found, however, that the infusion of activin A following LPS injection led to a 66% and 81% decrease in average microglial proliferation in the DG and pPV, respectively, when compared to the DG and pPV of vehicle-treated LPS control animals (Fig. 5B, 5C). We also found that infusion of activin A in LPS-injected animals led to a 35% and 40% decrease in the average total microglial populations in the DG and pPV, respectively, compared to the DG and pPV of animals that received LPS but no activin A (Fig. 5D). Our results confirm that activin A has potent anti-inflammatory effects, inhibiting microglial proliferation and reducing the total microglial population following LPS-stimulated local inflammation.

### Activin A Directly Regulates the Number and Activation of Microglia In Vitro

**Figure 6:**
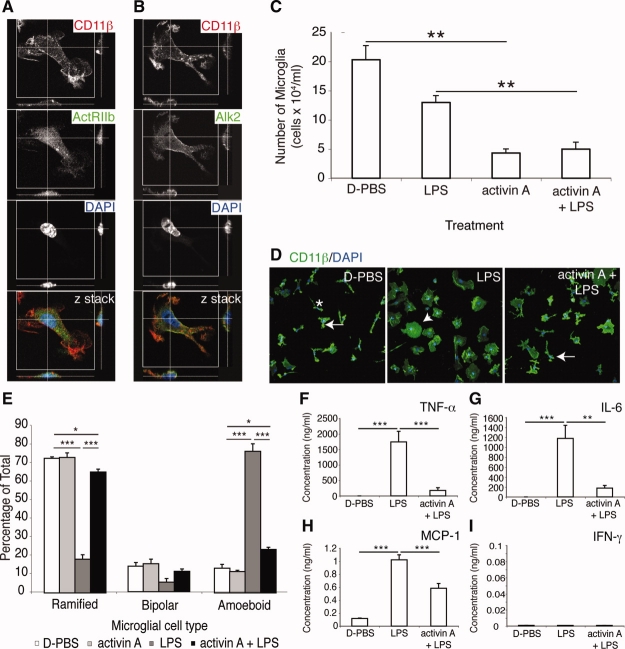
Activin A has direct anti-inflammatory effects on microglia in vitro. **(A, B):** Confocal z stack analysis revealed that CD11β^+^ microglia expressed the **(A)** type II receptor, ActRIIb, and the **(B)** type I receptor, Alk2, on the cell surface. **(C):** Total viable microglial counts showed that activin A decreases the microglial population in the presence and absence of LPS compared to control-treated microglial cells (LPS or D-PBS alone). **(D):** Microglial cells stimulated with LPS exhibited amoeboid (activated) morphology (arrowhead) compared to microglial cells treated with D-PBS control that exhibited ramified (resting) morphology (arrow). Microglial cells that were pretreated with activin A prior to LPS stimulation exhibited ramified (resting) morphology (arrow) comparable to microglial cells treated with D-PBS control. There were also microglial cells that exhibited bipolar morphology (asterisk). **(E):** Quantification of microglial cell subtypes revealed that stimulation with LPS (*n* = 3 experiments, light gray bars) increased the acquisition of amoeboid morphology, indicating microglial activation, compared to control-treated microglia (*n* = 3 experiments, white bars) exhibiting ramified (resting) morphology. Treatment of microglial cells with activin A (*n* = 3 experiments, dark gray bars) reversed the effects of LPS as groups treated with LPS and activin A showed similar levels of ramified (resting) microglial cells and amoeboid (activated) microglial cells as seen in microglia that were not stimulated with LPS. Treatment with activin A alone (*n* = 3 experiments, black bars) had no effect on microglial states compared to control-treated cells. There was also no change in microglial cells exhibiting bipolar morphology. **(F–I):** LPS stimulates release of **(F)** TNFα, **(G)** IL-6, and **(H)** MCP-1 but not **(I)** IFN-γ from cultured microglial cells, all of which are significantly inhibited by pretreatment with activin A. Values are mean ± SEM. ∗, *p* < .025; ∗∗, *p* < .005; ∗∗∗, *p* < .0005 (independent two-sample *t* test with Bonferroni correction). Abbreviations: ActRIIb, activin receptor IIb; Alk2 (ActRI), activin-like kinase receptor 2; DAPI, 4′,6-diamidino-2-phenylindole; D-PBS, Dulbecco's phosphate buffered saline; INFγ, interferon-γ; IL-6, interleukin-6; LPS, lipopolysaccharide; MCP-1, monocyte chemoattractant protein-1; PBS, phosphate buffered saline; TNFα, tumor necrosis factor-α.

We next set out to confirm that the effects of activin A on microglia were cell-autonomous. For activin A to directly regulate inflammation, microglia would have to possess both type I and type II activin receptors [31]. Using immunocytochemistry, we confirmed that cultured primary microglial cells, labeled for CD11β, expressed the type II receptor, ActRIIb (Fig. 6A), and the type I receptor Alk2 (ActRI) (Fig. 6B).

To determine whether activin A can regulate microglia numbers directly, cultured primary microglial cells were treated with vehicle, LPS, activin A plus LPS, or activin A alone for 24 hours. LPS had no statistically significant effect on the number of microglial cells compared to (D-PBS)-treated microglial cells (Fig. 6C). However, microglial cells treated with activin A with and without LPS showed an 80% and 62% decrease in average total cell numbers, respectively, when compared to microglial cells that received LPS alone or D-PBS alone (Fig. 6C). These results support the in vivo data and suggest that activin A alters microglial numbers by acting directly on the cells to inhibit TLR4 signaling pathways.

Activation of microglial cells in vitro by LPS is characterized by morphological changes from ramified morphology (arrow, Fig. 6D), indicating resting state, to bipolar morphology (asterisk, Fig. 6D), indicating transition state, and then into amoeboid morphology (arrowhead, Fig. 6D), indicating activation state [32]. Therefore, we next investigated whether the addition of activin A directly altered the morphological states of microglia. We found that the addition of activin A alone did not result in any significant changes in the percentage of cells exhibiting ramified, bipolar, or amoeboid morphology compared to (D-PBS)-treated control cultures (Fig. 6E). We found that addition of LPS resulted in a 461% increase in microglia cells exhibiting amoeboid-activated morphology and a concurrent 75% decrease in ramified resting microglia (Fig. 6E). Treatment of microglial cells with activin A prior to stimulation with LPS resulted in a 261% increase in microglial cells exhibiting ramified resting morphology and a concurrent 69% decrease in amoeboid-activated microglial cells compared to LPS-treated microglial cells (Fig. 6E), indicating that activin A is capable of directly reversing or preventing microglial activation by LPS. The levels of ramified (resting), bipolar, and amoeboid (activated) microglial cells in activin A plus LPS-treated cultures were comparable to that observed in (D-PBS)-treated control cells (Fig. 6E).

Since we observed changes in microglial morphology, we next set out to determine whether activin A altered cytokine release from microglial cells. We found that stimulation of microglial cells with LPS resulted in significant increases in the expression of several pro-inflammatory cytokines including tumor necrosis factor-α (TNFα) (Fig. 6F), interleukin (IL)-6 (Fig. 6G), and monocyte chemoattractant protein (MCP)-1 (Fig. 6H). Pretreatment of microglial cells with activin A prior to stimulation with LPS resulted in a significant reduction in the release of TNFα (Fig. 6F), IL-6 (Fig. 6G), and MCP-1 (Fig. 6H). The levels of the cytokines interferon (IFN)-γ (Fig. 6I), IL-10 (data not shown), and IL-12p70 (data not shown) were below the levels of detection, indicating that the microglia do not release significant amounts of these cytokines (IFN-γ, IL-10, or IL-12p70) in response to LPS stimulation. Combined with the study conducted by Sugama et al., which showed that activin A reduced cytokine expression in LPS-treated microglia [33], our results show that activin A directly inhibits microglial proliferation, microglial activation, and microglial release of pro-inflammatory cytokines.

### Activin A Effects on Neurogenesis Are Secondary to Its Effects on Inflammation

**Figure 7:**
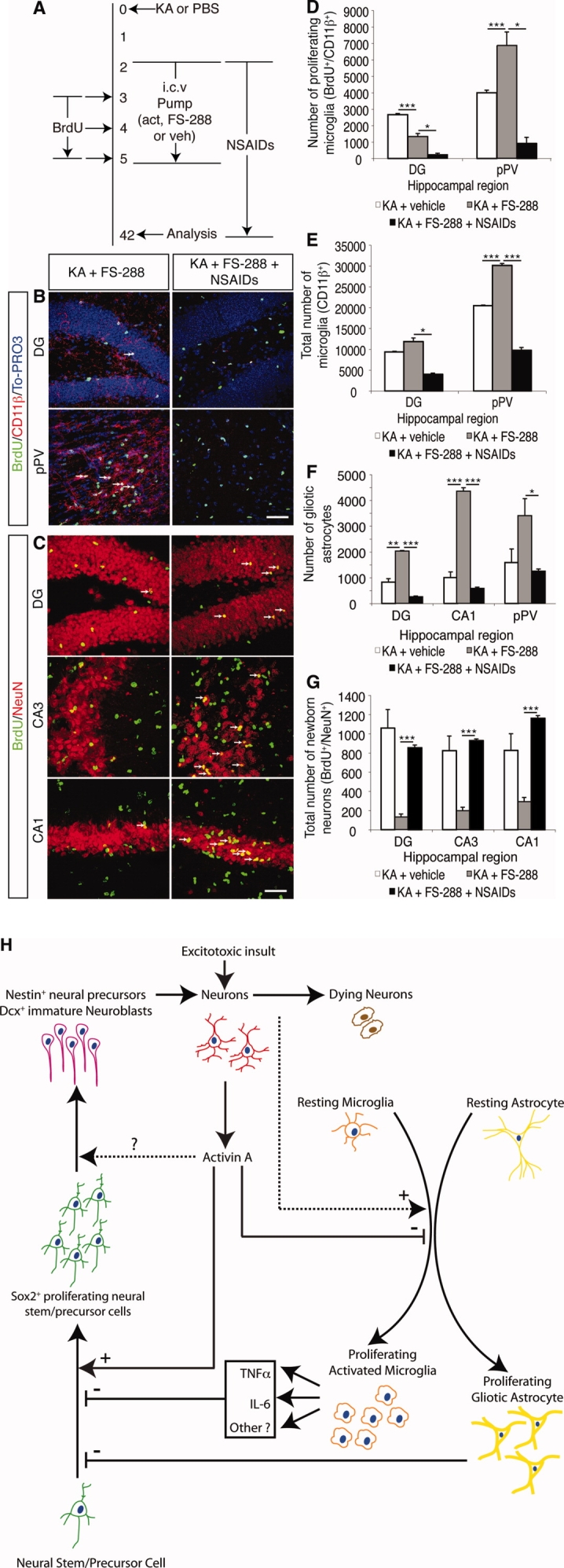
NSAIDs inhibit microglial proliferation, inhibit gliosis, and restore neurogenesis in (FS-288)-treated KA-injected animals. **(A):** Experimental timeline. Some animals received NSAIDs beginning on day 2, prior to implantation of osmotic micropumps, and ending on day 42 when tissue was harvested. **(B):** Immunohistochemistry and confocal analysis of proliferating microglial cells in (FS-288)-treated, KA-injected animals that did or did not receive NSAIDs. Scale bar = 50 μm. **(C):** Immunohistochemistry and confocal analysis of newborn neurons (arrows) in the DG, CA3, and CA1 regions in (FS-288)-treated, KA-injected animals that did or did not receive NSAIDs. Scale bar = 50 μm. **(D, E):** Quantification of microglial populations revealed that NSAIDs in (FS-288)-treated, KA-injected animals (*n* = 5, black bars) inhibited **(D)** microglial proliferation and reduced **(E)** the total microglial population in the DG and pPV compared to (FS-288)-treated, KA-injected animals that did not receive NSAIDs (*n* = 5, gray bars). **(F):** Quantification of gliosis, defined as the number of proliferating astrocytes exhibiting gliotic morphology, revealed that NSAID treatment of (FS-288)-treated, KA-injected animals (*n* = 5, black bars) decreased the extent of gliosis in the DG, CA1, and pPV compared to (FS-288)-treated, KA-injected animals that did not receive NSAIDs (*n* = 5, gray bars). **(G):** Quantification showed significant recovery of neurogenesis in the DG, CA3, and CA1 regions of (FS-288)-treated, KA-injected animals that received NSAIDs (*n* = 5, black bars) compared to (FS-288)-treated, KA-injected animals that did not receive NSAIDs (*n* = 10, gray bars). **(H):** Local activin A expression following injury acts as an anti-inflammatory, inhibiting gliosis and microglial activation while promoting neurogenesis. Neurodegeneration activates microglia, possibly in part through activation of TLR2 and TLR4 receptors (see Discussion), and also directly and/or indirectly leads to a gliotic response by astrocytes. Microglial activation leads to release of pro-inflammatory cytokines, including TNF-α and IL-6. Cytokines inhibit neural stem/precursor cell proliferation and, consequently, neurogenesis. However, increased activin A expression from surviving neurons is a potent anti-inflammatory agent that inhibits proliferation and activation of microglia and either directly and/or indirectly inhibits the gliotic response by astrocytes. This in turn is permissive for neurogenesis. Activin A also regulates neurogenesis by stimulating neural stem/precursor proliferation, leading to increased number of immature neuroblasts and, ultimately, increased neurogenesis. It is uncertain whether activin A also alters differentiation of neural stem/precursor cells. This model does not exclude the possibility that other transforming growth factor-β/bone morphogenetic protein molecules act in concert with activin A; however, their actions would also be, at least in part, anti-inflammatory (see Discussion). The fact that activin A is expressed by neurons raises the possibility of an internal response system, where injured neurons signal for anti-inflammatory action. This model is of relevance for understanding not only postinjury response within the central nervous system but also the environment of neurodegenerative disease. Values are mean ± SEM. ∗, *p* < .025; ∗∗, *p* < .005; ∗∗∗, *p* < .0005 (independent two-sample *t* test with Bonferroni correction). Abbreviations: BrdU, bromodeoxyuridine; Dcx, doublecortin; DG, dentate gyrus; FS-288, follistatin-288; IL-6, interleukin-6; KA, kainic acid; NeuN, neuronal nuclei; NSAIDs, nonsteroidal anti-inflammatory drugs; PBS, phosphate buffered saline; pPV, posterior periventricular area; TLR, toll-like receptor; TNFα, tumor necrosis factor-α.

Our data strongly suggest that activin A is a potent anti-inflammatory in the CNS (Figs. 5, 6). Inflammation has been shown to inhibit neurogenesis [34, 35]. This led us to hypothesize that the essential role of activin A in neurogenesis after cell loss is secondary to its inhibition of inflammation. Therefore, since FS-288 binds to activin A and thereby inhibits activin A, we reasoned that the inhibition of neurogenesis by FS-288 may result from inhibition of the anti-inflammatory effects of activin A. In that case, neurogenesis should proceed whether activin A is blocked by follistatin, providing inflammation is suppressed in some other way. To test this hypothesis, we investigated whether nonsteroidal anti-inflammatory drugs (NSAIDs), indomethacin and minocycline, would reverse the effects of FS-288 on inflammation and neurogenesis after KA-induced excitotoxic neurodegeneration (Fig. 7A).

We first investigated whether administration of NSAIDs to animals that received FS-288 after KA injection had significant anti-inflammatory effects as expected. As shown in Figure 7, NSAID treatment resulted in 83% and 87% reduction in the average number of proliferating microglial cells in the DG and pPV, respectively (Fig. 7B, 7D) and a reduction of 66% and 68% in the average total microglial population in the DG and pPV, respectively (Fig. 7E), compared to KA-injected (FS-288)-treated animals that did not receive anti-inflammatory agents (Fig. 7B, 7D, 7E). Similarly, gliosis was reduced by NSAID treatment (Fig. 7F). Specifically, the average number of proliferating astrocytes that displayed gliotic morphology in the DG, CA1, and pPV was 87%, 87%, and 63% less than that observed in KA-injected, (FS-288)-treated animals that did not receive NSAIDs (Fig. 7F). These results confirm that, in the KA-injured hippocampus, the inflammatory effects of FS-288 are reversed by NSAIDs.

To determine whether inhibition of inflammation in animals that received a KA injection and FS-288 infusion induces recovery of neurogenesis, tissue was analyzed for the number of newly generated neurons in the DG, CA3, and CA1 (Fig. 7C). We first confirmed that i.c.v. infusion of FS-288 almost abolishes neurogenesis after neurodegeneration (compare the gray bars with the white bars in Fig. 7G). FS-288 binds to activin A and therefore blocks the activin A that is expressed in the brain from having any effect. This data confirms that, in the absence of activin A, neurogenesis does not proceed after neurodegeneration. The effect of FS-288 on neurogenesis was quite profound.

When we added a nonsteroidal anti-inflammatory agent together with FS-288, the neurogenesis was restored to levels observed when endogenous activin A is not blocked (compare the black bars with the white bars in Fig. 7G). Indeed, the average number of newly generated neurons in the DG, CA3, and CA1 was increased by 547%, 371%, and 299%, respectively, in animals that received NSAIDs compared to the DG, CA3, and CA1 regions of KA-injected (FS-288)-treated animals that did not receive NSAIDs (Fig. 7G). The extent of neurogenesis in animals that received NSAIDs was comparable to the extent of neurogenesis that we observed in the DG, CA3, and CA1 of vehicle-treated KA-injected animals (Fig. 7G).

In summary, neurogenesis after neurodegeneration requires expression of activin A (Fig. 7H). Activin A signaling stimulates neurogenesis after neurodegeneration by inhibiting inflammatory and gliotic mechanisms, which would otherwise negatively regulate neural stem/precursor cell populations in the adult hippocampus. If activin A is blocked, neurogenesis can be completely restored by adding a nonsteroidal anti-inflammatory agent, even in the absence of active activin A. Thus, neurogenesis will proceed after neurodegeneration, providing an anti-inflammatory agent is present, whether it be activin A or, in its absence, an exogenously administered anti-inflammatory agent.

## DISCUSSION

We provide evidence for a centrally derived anti-inflammatory response after acute excitotoxic neurodegeneration that is required for neurogenesis to proceed in vivo. Several studies have demonstrated that activin A is expressed by neurons following an acute excitotoxic insult to the CNS [11-13, 19]. Our data demonstrate that activin A exerts potent anti-inflammatory effects in the CNS by suppressing microglial numbers, microglial activation, and pro-inflammatory cytokine release. Activin A also directly or indirectly suppresses gliosis. Consistent with this, we found that, after KA-induced neurodegeneration, administration of FS-288, an activin A antagonist, in the CNS in vivo profoundly increased microglial numbers and gliosis compared to that induced by KA alone. This (FS-288)-induced inflammatory response, in turn, led to a profound reduction in neurogenesis, an effect that was reversed by the administration of NSAIDs. Thus, FS-288 exerts its effects on neurogenesis, at least in part through blockade of an intrinsic anti-inflammatory mechanism in the CNS. Our results therefore demonstrate that activin A, which is expressed by neurons, is capable of inducing neural stem/progenitor cell proliferation, leading to increased neurogenesis, through a potent anti-inflammatory action in vivo (Fig. 7H).

It is increasingly thought that Parkinson's disease, Alzheimer's disease, spinal cord injury, seizures, and excitotoxicity may involve a microglial response that is regulated by TLR4 signaling [26, 29, 36–38]. TLR4 is also activated by LPS [26, 29, 36, 38, 39]. We showed that activin A blocks LPS-mediated microglial activation in vivo and in vitro. We also observed that activin A impaired inflammation following KA-induced neurodegeneration. Given the evidence for TLR4 activation in degenerative conditions [26, 29, 36, 37], it is feasible that the effect of activin A on inflammation following excitotoxic neurodegeneration may also result from inhibition of TLR4-mediated signaling in microglia. Regardless, although activin A may not be expressed exclusively by neurons in the CNS, our study suggests that activin A expression by neurons is part of an intrinsic overall centrally derived anti-inflammatory response to excitotoxic neurodegeneration that regulates neurogenesis in the CNS. This raises the possibility of an internal response system, where injured neurons signal for anti-inflammatory action. This is of relevance for understanding not only postinjury response within the CNS but also the environment of neurodegenerative disease.

The TGFβ superfamily consists of two branches, the bone morphogenetic protein (BMP)/growth differentiation factor (GDF) branch and the TGFβ/activin branch. Molecules in the BMP/GDF branch have been shown to inhibit neurogenesis and stimulate the formation of new astrocytes [40–42]. Some studies suggest that ligands in the TGF/activin branch, TGFβ1, TGFβ2, and TGFβ3, have neurogenic effects [43], whereas contrasting studies suggest that inhibition of TGFβ ligands enhances neurogenesis in the adult hippocampus [44, 45]. Furthermore, TGFβ signaling effectors such as smad4, smad1/5/8, and smad2/3 have been shown to play a role in neurogenesis [43]. Clearly, the effects of TGFβ ligands on neurogenesis are complex.

An important conclusion from our work that may explain this complexity is that the effect of a ligand on neurogenesis will depend on the inflammatory state of the tissue, the region of the brain being analyzed, and more particularly, the indirect effects of the ligand via actions on other cell types that in turn regulate neurogenesis. In other words, it appears that interactions in the cellular microenvironment critically define the effect of activin A on neurogenesis.

Our data showed that administration of activin A to the uninjured hippocampus increased neural stem/precursor cell proliferation and, consequently, neurogenesis (Fig. 2D, 2F). Indeed, neurogenesis induced by 3 days of activin A treatment in the intact brain was sufficient to increase the total neuron population in the hippocampus. The increased number of neurons resulted from changes in proliferation of Sox2-positive neural stem cells [21] and nestin-positive precursor cells [22], an effect that led to increased numbers of Dcx expressing neuroblasts and neurogenesis. This suggests that activin A can have a direct effect on neurogenesis in the animal in the absence of KA-induced injury. However, activin A expression is low in the intact brain. Therefore, activin A is unlikely to play a role in neurogenesis in the absence of injury. We confirmed this when we gave an i.c.v. infusion of FS-288 (a high-affinity antagonist of activin A) into the intact brain and found that it did not block baseline neurogenesis. Clearly, activin A can stimulate neurogenesis but it is not required for neurogenesis in the absence of injury (Fig. 2D, 2F). So whereas activin A may stimulate neurogenesis directly, it is of minimal physiological relevance in the intact brain.

We also observed that KA-induced neurodegeneration was followed by extensive proliferation of Sox2-positive neural stem cells [21] and nestin-positive neural precursor cells. This was followed by neurogenesis and recovery of the total neuron numbers in the hippocampus (Fig. 2E). We confirmed that KA-induced neurodegeneration leads to expression of activin A. FS-288, a high-affinity activin A antagonist, profoundly impaired precursor cell proliferation and neurogenesis, an effect that was entirely reversed by the administration of NSAIDs (Fig. 7). Thus, progression of neurogenesis after neurodegeneration requires the presence of an anti-inflammatory agent, either endogeneously expressed activin A or, in its absence, an exogenously administered anti-inflammatory agent. Given the large increase in the number of Sox2-positive and nestin-positive cells and Dcx-positive migrating neuroblasts observed after KA, it seems that the anti-inflammatory actions of activin A in turn permit the proliferation of multipotent and self-renewing NSCs [21] and the subsequent proliferation and/or differentiation of neural precursor cells, which in turn lead to neurogenesis.

A physiologically important question is whether endogenously expressed activin A is required for neurogenesis (beyond its postinjury inhibition of inflammation) in the neurodegenerating brain? Endogenously expressed activin A may directly stimulate neurogenesis after neurodegeneration (because it stimulated neurogenesis directly in the intact brain, Fig. 2). However, although this would be important in terms of facilitating the neurogenesis after neurodegeneration, the data shown in Figure 7G make it clear that a direct effect of activin A on stem/precursor cells is not an essential requirement for neurogenesis after neurodegeneration. We know this because we observed that neurogenesis proceeds following KA-induced degeneration when activin A is blocked, provided that a nonsteroidal anti-inflammatory drug is also present. We therefore conclude that the essential role of centrally derived activin A is an anti-inflammatory response that is permissive for neurogenesis. Any direct role that activin A exerts directly on neurogenesis would facilitate regeneration but is not essential for it.

Thus, activin A exerts its critical effects on neurogenesis through inhibition of the replication and activation of inflammatory cells and through inhibition of cytokine release from these cells (Figs. 5, 6). When activin A is blocked, an exogenously administered anti-inflammatory is sufficient to restore its effect (Fig. 7). This suggests that molecules other than activin A are primarily responsible for driving the proliferation of stem/precursor cells although activin A may also directly assist this process. The essential role of activin A, however, is to provide an environment that is permissive for neurogenesis. This has significant implications for future research into mechanisms of regeneration in neurodegenerative diseases and for the development of treatments.

Although data from previous in vitro studies have suggested that TGFβ ligands, including activin A, induce the differentiation of neural precursors into astrocytes, while also inhibiting proliferation of astrocyte lineage cells [15, 40, 46], we found no evidence for such an effect of activin A in the adult CNS in vivo. This again points to the importance of the cellular niche in determining the effects of a ligand in vivo. Activin A infusion did not lead to a detectable change in the number of newly generated astrocytes in brains that did not receive KA (Fig. 3). Furthermore, activin A infusion also did not lead to a change in glial cell proliferation in brains after KA-induced neurodegeneration. Our data, however, is consistent with the role of activin A in regulating gliosis, either directly or indirectly, since FS-288 infusion greatly increased astrocyte proliferation and gliosis after KA-induced neurodegeneration (Fig. 3). Microglia can regulate gliosis [47]; therefore, it is possible that the effects of FS-288 on gliosis we observed in vivo occur secondarily to its pro-inflammatory effects on microglia. Given that glia can regulate neural stem/precursor cell proliferation and neurogenesis [48, 49], the extent of gliosis will, in turn, regulate neurogenesis. In addition, it has been established that inflammatory cytokines that are expressed by activated microglia alone are sufficient to directly inhibit neurogenesis [34].

We observed that other TGFβ superfamily ligands such as BMP1, BMP2, BMP5, and BMP7 were also expressed in the CNS after KA-induced neurodegeneration (supporting information Table 1). There are reports that, in addition to its high-affinity antagonism of activin A, FS-288 may also have antagonist activity against other TGFβ superfamily molecules, especially BMP4, BMP7, and TGFβ1 [50] and osteogenic protein-1 [51]. This raises the possibility that FS-288 in our study may mediate some of its effects through binding other BMP and TGFβ molecules, in addition to binding to activin A. However, we showed that NSAIDs reversed the inhibitory effects of FS-288 on neurogenesis. Therefore, if blockade of other specific BMP molecules contributes to the inhibitory effects of FS-288 on neurogenesis, then those specific BMP molecules are also working in concert with activin A, at least in part, to impair the inflammatory response after KA-induced neurodegeneration.

Activin A appears to have diverse roles in tissue repair, fibrosis, and inflammation outside the CNS [52]. Our study shows that activin A, which is released by neurons, is an anti-inflammatory agent in the CNS. It has also been reported that, outside the CNS, activin A has anti-inflammatory effects on myeloblasts, monocytic M1 cells, B lymphoid cell line, and hepatoma cell lines [52–54]. In surprising contrast to this, a recent study [55] suggests that follistatin, administered i.p., has anti-inflammatory effects in the peripheral circulation in response to an i.p. injection of LPS. The mechanisms by which follistatin exerted these anti-inflammatory effects in the peripheral circulation is unclear. The peripheral actions of activin A and follistatin on inflammation are clearly complex and unraveling this will require a broad understanding of TGFβ superfamily effects, both on specific leukocyte cell types and on the complex cellular interactions that underlie the peripheral inflammatory response in vivo.

Although increasing evidence suggests that neurogenesis is functionally important [1, 2], the role of neurogenesis in normal brain function and disease remains to be fully elucidated. Evidence suggests that neurogenesis is impaired in neurodegenerative diseases, an effect that could contribute to the overall loss of neurons and cognitive decline [3]. Recent literature has demonstrated that inflammation inhibits neurogenesis in the adult hippocampus [34, 35]. Our study is the first to demonstrate an endogenous mechanism that is required for neurogenesis following acute excitotoxic neurodegeneration that exerts its effect by inhibiting the detrimental effects of inflammation. Although we have shown that the anti-inflammatory effect of activin A, and perhaps other TGFβ superfamily molecules, is permissive for neurogenesis, it is possible that this anti-inflammatory effect would also contribute to the reported neuroprotective effects of TGFβ superfamily molecules [12, 19]. Thus, we suggest that activin A is a pivotal molecule at the nexus between neurodegeneration, inflammation, and neural regeneration.

Chronic neurodegenerative diseases are characterized by chronic inflammation and reduced neurogenesis [3, 17, 56]. Our study now raises the question of whether expression of an endogenous brain-derived anti-inflammatory response is impaired in chronic neurodegenerative diseases. If so, this would explain why a regenerative response is seen after acute neurodegeneration but not in chronic neurodegenerative diseases. Recent studies have reported deficient TGFβ signaling in neurodegenerative disorders including Alzheimer's disease [57]. These conditions are also characterized by increased inflammation [58–60]. It is, therefore, quite possible that an altered endogenous anti-inflammatory response may contribute to the observed inflammation in these chronic diseases, an effect that would, in turn, contribute to reduced neuronal survival and reduced regeneration that lead to their inevitable pathological outcomes.

## DISCLOSURE OF POTENTIAL CONFLICTS OF INTEREST

The authors indicate no potential conflicts of interest.
