# Supplementary figures and images for: Activin A Is Essential for Neurogenesis Following Neurodegeneration
Source: Stem Cells. 2009 Jun;27(6):1330–46. doi: 10.1002/stem.80 (PMC2733378; doi:10.1002/stem.80)

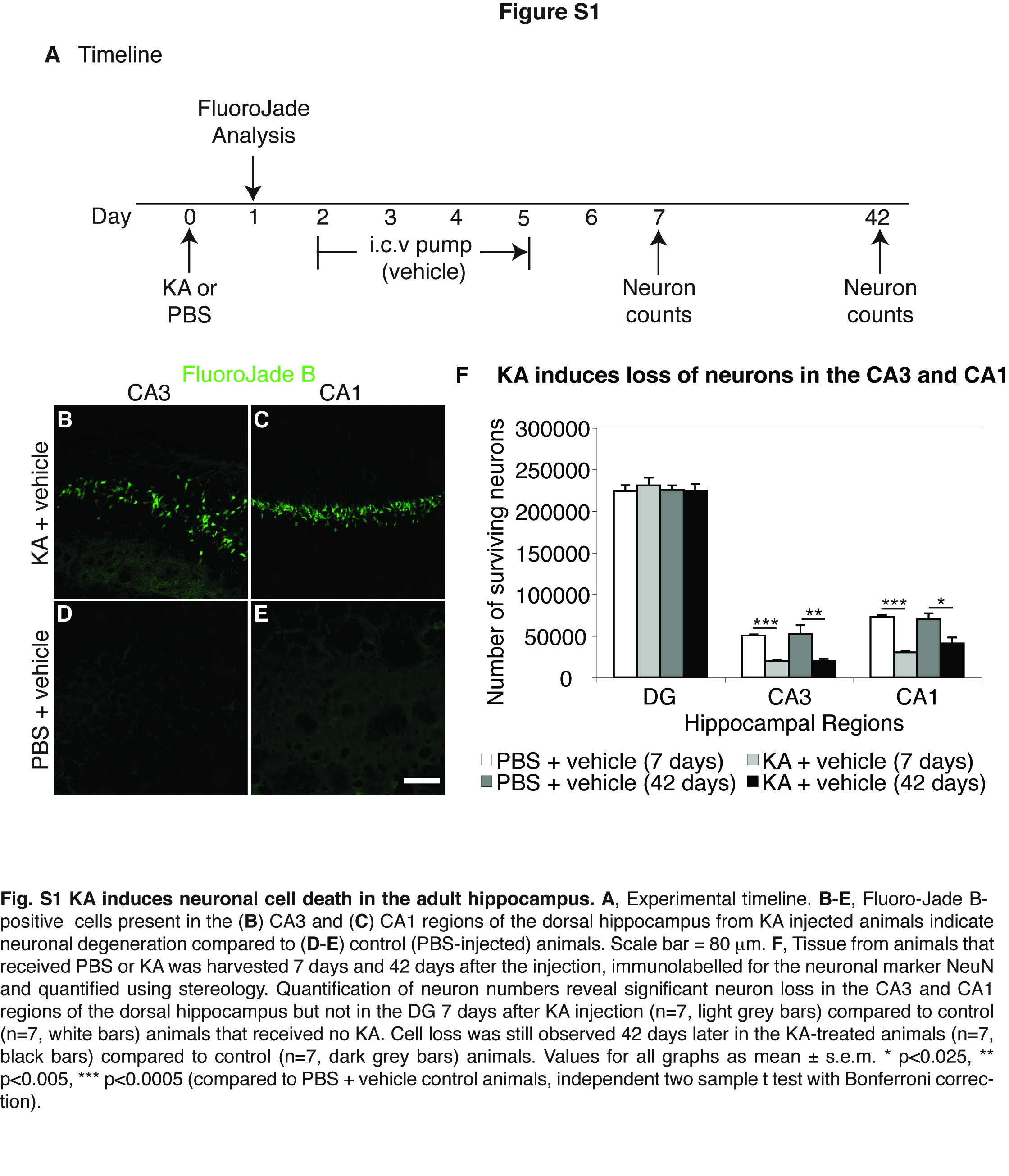

Supplement: Supplementary file 1 [file stem0027-1330-SD1.tif]

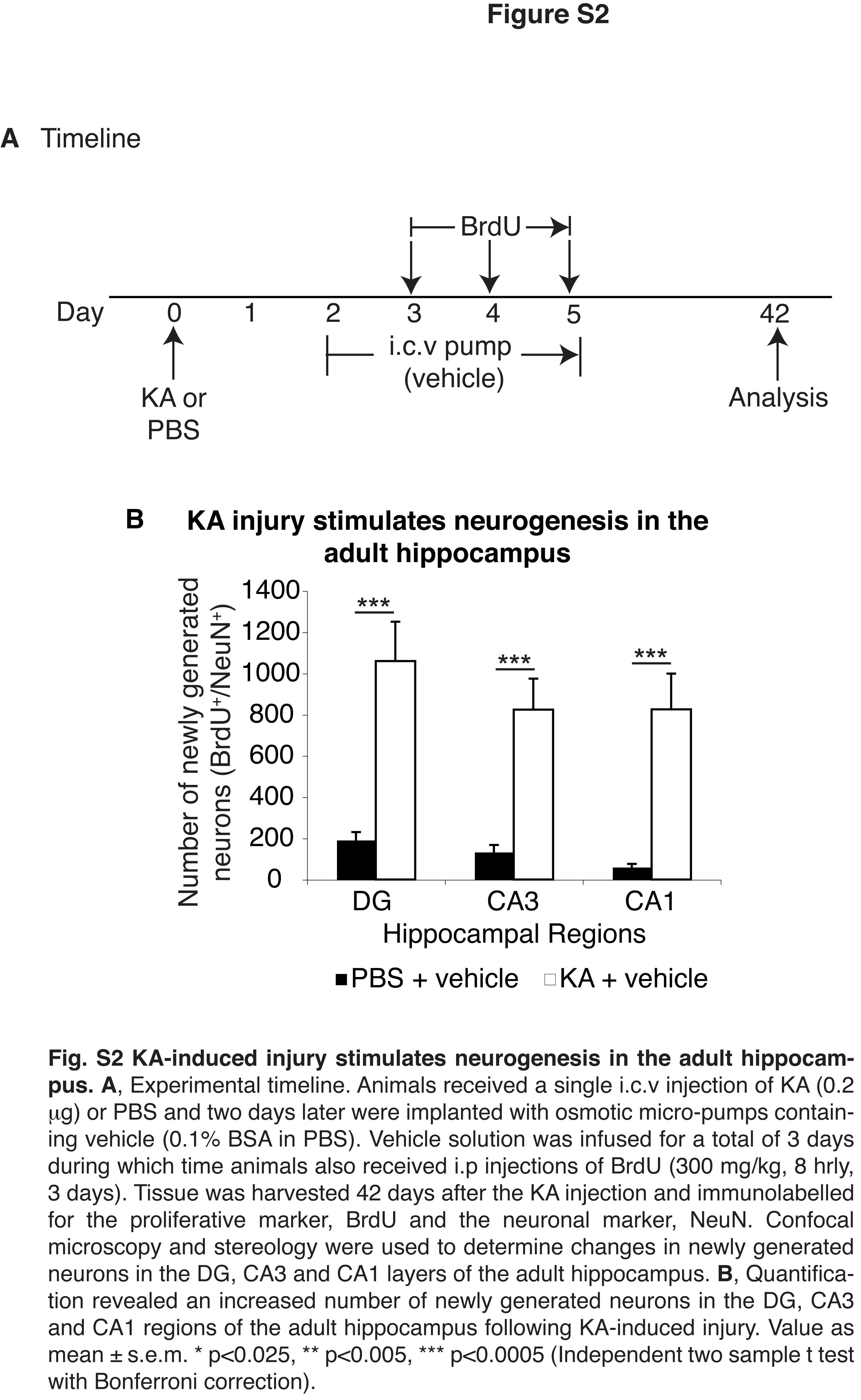

Supplement: Supplementary file 2 [file stem0027-1330-SD2.tif]

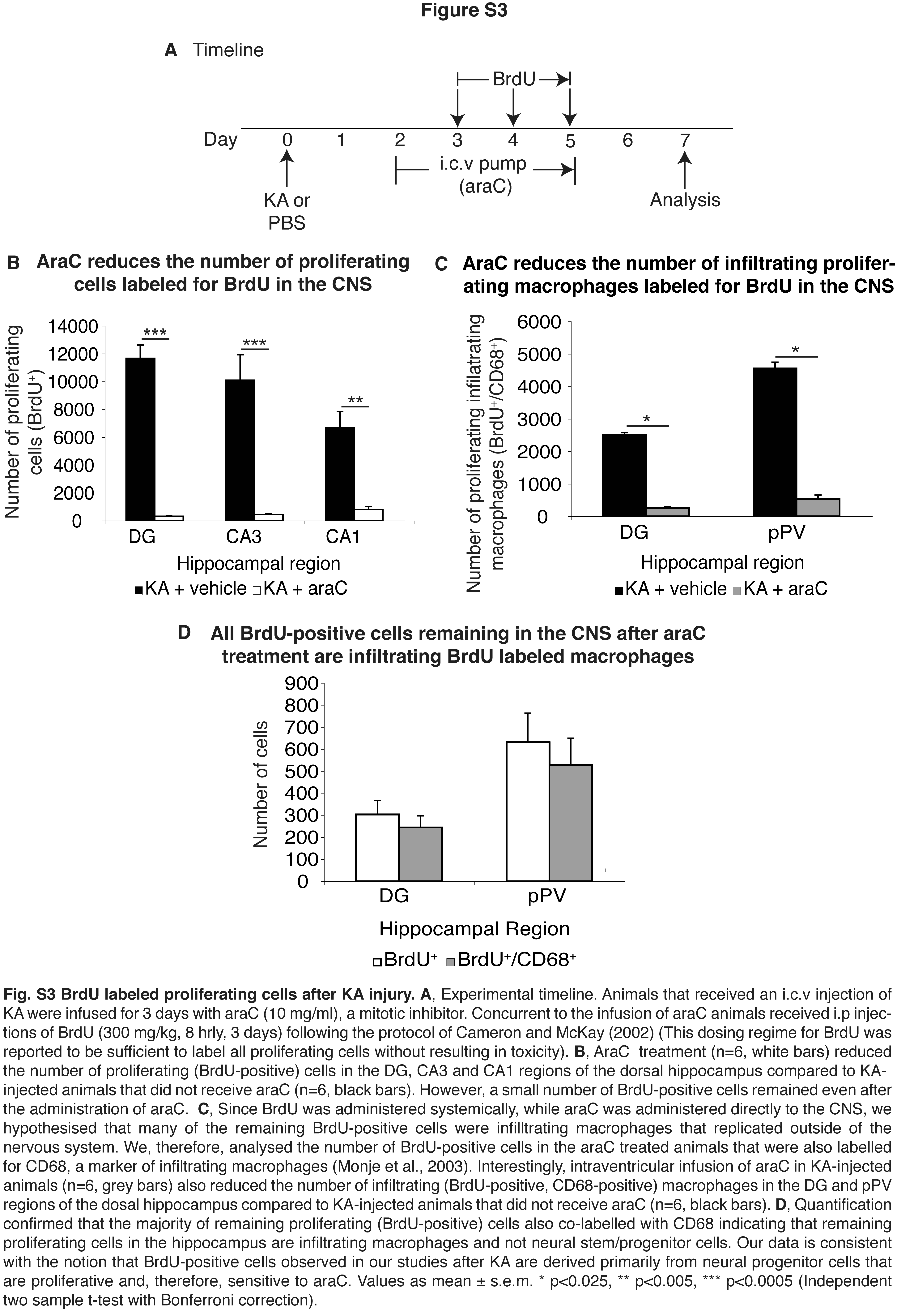

Supplement: Supplementary file 3 [file stem0027-1330-SD3.tif]

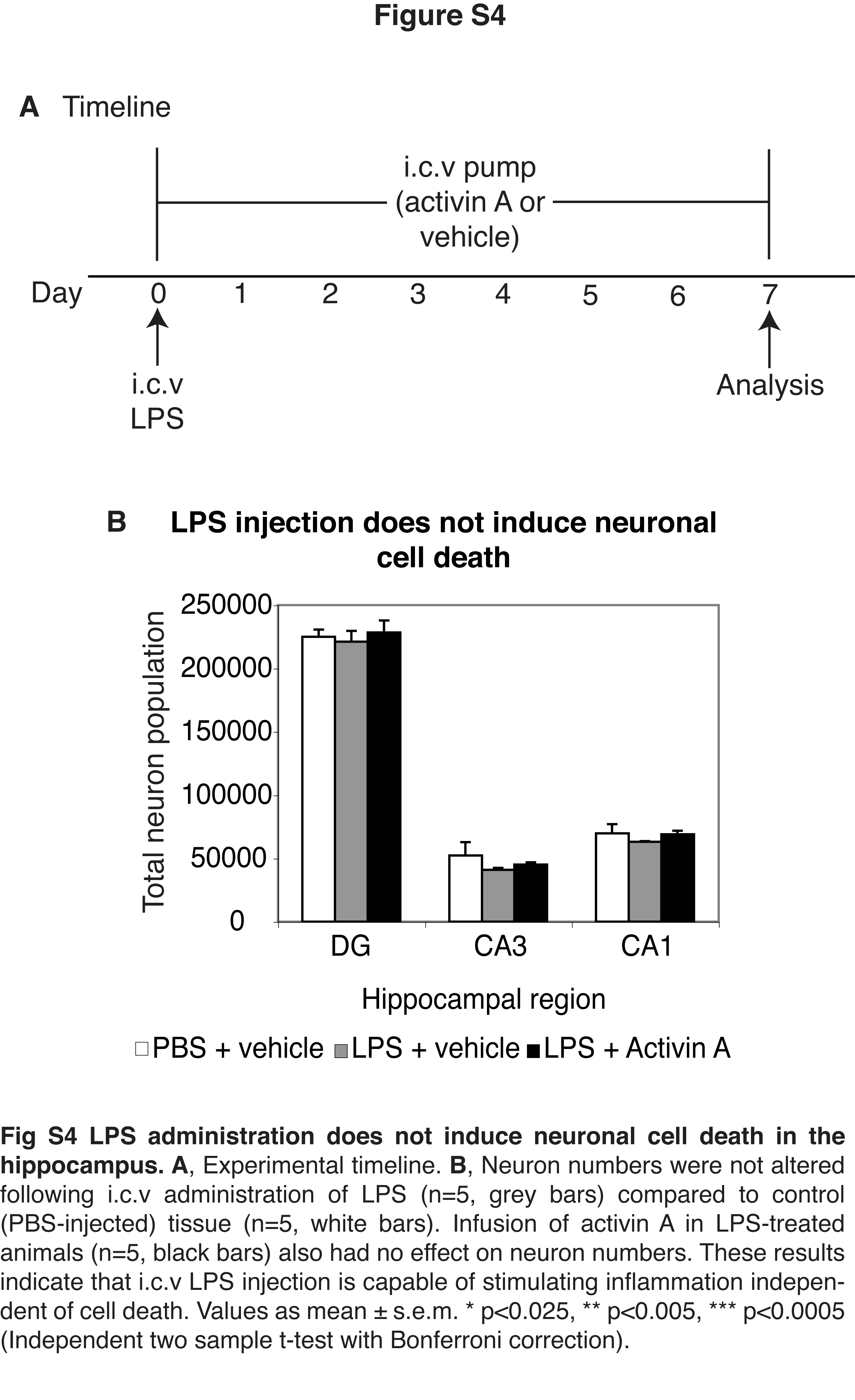

Supplement: Supplementary file 4 [file stem0027-1330-SD4.tif]
