# Supplementary material for: Activin A Is Essential for Neurogenesis Following Neurodegeneration
Source: Stem Cells. 2009 Jun;27(6):1330–46. doi: 10.1002/stem.80 (PMC2733378; doi:10.1002/stem.80)
Supplement: Supplementary file 5 [file stem0027-1330-SD5.doc]

**Table I. Changes in the level of mRNAs encoding potential neurogenic growth factor following treatment of the hippocampus with KA using real-time RT-PCR. Panel A**, Experimental timeline. Animals received a single i.c.v injection of KA or PBS. Osmotic micro-pumps containing vehicle were implanted 48 hrs later. Hippocampal lobes ipsilateral to the site of injection and pump implantation were harvested 6 hrs after pump implantation. The change in expression of mRNA was examined using quantitative real-time RT-PCR (refer to Supplemental methods). **Panel B**, Real time RT-PCR revealed that the injection of KA resulted in the upregulation of mRNAs encoding ligands of the BMP/GDF branch including BMP1/2/5/7, and ligands of the TGF/activin branch including activin βA and TGFβ1 . BMP1, BMP2, BMP5 and BMP7 were upregulated following KA by 2, 2.5, 3, and 7 fold, respectively. However, activin βA was upregulated by approximately 24 fold compared to tissue from PBS treated animals. Thus, activin A is a major TGFβ superfamily ligand with increased expression after KA-induced neurodegeneration in the hippocampus. The data are shown as mean ± s.e.m. * p<0.05 in the one-way ANOVA followed by post-hoc multiple comparison Tukey-Kramer test.

**PANEL A**

**PANEL B**
