# Supplementary material for: Activin A Is Essential for Neurogenesis Following Neurodegeneration
Source: Stem Cells. 2009 Jun;27(6):1330–46. doi: 10.1002/stem.80 (PMC2733378; doi:10.1002/stem.80)
Supplement: Supplementary file 7 [file stem0027-1330-SD7.doc]

Supplemental Material and Methods For

Activin A is essential for neurogenesis following neurodegeneration

Abdipranoto-Cowley A, Park JS, Croucher D, Daniel J, Henshall S, Galbraith S, Mervin K, and Vissel B

**Supplemental Materials and Methods**

Stereology

*Estimation of the total neuron populations using the StereoInvestigator 7*. Every 6th section was prepared from coronal brain slices. A total of 5 sections were sampled with antero-posterior positions from bregma of -1.34, -1.58, -1.82, -2.06, and -2.3 mm. For assessments of neuron numbers, counts were performed using the optical fractionator probe in Stereo Investigator 7 (Microbrightfield). Design parameters were determined prior to commencement of the study. A minimum 21 sampling sites were sampled per section on a grid size of 84 μm x 60μm. At each sampling site a counting frame size of 50 μm x 50 μm, guard zones of 5 μm and a disector height of 10 μm were used.

*Estimation of double-labelled cell populations using confocal microscopy and the optical fractionator design method.* For counts of cells that were double immunolabelled for BrdU and other cell markers, unbiased stereology and confocal microscopy were used. The entire region of interest between bregma positions AP -1.34 mm and AP -2.3 mm was sampled and cells on the selected sections were counted. A counting frame size of 65 μm x 65 μm, guard zones of 5 μm and a disector height of 10 μm were used. The number of cells sampled was converted into the estimated total number of cells in the region of interest using the optical fractionator design method. The number of cells sampled in double immunolabelling counts using confocal microscopy and unbiased stereology was converted into the total estimated number of particles in the region of interest using the optical fractionator design method:

(Dorph-Petersen et al, 2001)

where:

N is the total number of cells estimated

*bsf* is the block sampling fraction

*ssf* is the section sampling fraction

*hsf* is the height sampling fraction

*h* is the constant height of the optical disectors

*t* is the local section thickness

∑ Q^-^ is the total count of cells finally sampled by the optical disectors

Methodological considerations for stereology

*Experimental design.*

Design-based stereology requires that the entire structure be sampled with equal probability and that the actual sampling of cells use an unbiased selection for counting. Systematic-random sampling of sections from exhaustive section series encompassing the entire region of interest will allow access to the region of interest. Random section offset is used to reduce the chance for potential bias so that for any given subject, until a section is chosen at random, each and every cell must have the same statistical probability of getting sampled. However, in our study the cell distribution within the region of interest appeared to be homogenous, and therefore, a random section offset was not required.

In our study we implemented counting rules that could not be affected by shape, size, orientation and distribution of the object/particle. The probe we used to count was called the counting frame and was shown as a red and green probe representing rejection and acceptance lines, respectively. The counting rule used in this study was that the top of a particle was counted if it lay inside the counting frame and didn’t touch an exclusion line. However, when the top of a cell lay completely outside of the counting frame but touched an inclusion line, but not an exclusion line, then we also counted it.

*Effect size.* When performing stereological population estimates sources of variability include biological variability, which is the variability observed between animals, and the sampling error. Oversampling populations leads to confidence that the sampling error is significantly lower than the biological variability, in which case, the spread of the population estimates serve as a good indicator of biological variability. Therefore, we oversampled our populations by ensuring large sample sizes (between 5-12 animals per treatment group) and by counting the entire region of interest in each section, thereby oversampling within each section. The large effects of the treatments that we observed on the estimated number of cells made it evident that our population estimates were statistically robust.

Cell Culture

*Primary Cell culture.* Pregnant female C57Bl/6 mice were imported from the Animal Resources Centre (Perth, Australia). Briefly, cerebra taken from P1-P2 mouse pups were chopped, trypsinised (0.5% trypsin, 10 min, 37°C/5% CO_2_) and triturated. The cell suspension was washed in culture media (DMEM/F12 with 10% fetal bovine serum (FBS), 1 mM sodium pyruvate, 100 U/ml penicillin, 100mg/ml streptomycin) and cultured at 37°C/5% CO_2_ in poly-D-lysine (10 mg/ml in borate buffer)-coated 75 cm^2^ tissue-culture flasks. The media was changed once a week. At 10-14 days in vitro (DIV) microglial were shaken off the primary mixed glial cultures (150 rpm for 6 hrs) and plated onto PDL-coated glass coverslips or onto 24-well plates and grown in culture media for microglia (RPMI-1640 with 10% FCS, 1 mM L-glutamine, 1 mM sodium pyruvate, 50 mM 2-mercaptoethanol, 100 U/ml penicillin, 100 mg/ml streptomycin).

Analysis of mRNA expression changes for data shown in Supplementary tables I and II

*Sampling.* Animals received a single i.c.v injection of KA (0.2 μg) or PBS and were subsequently implanted with osmotic micro-pumps containing vehicle solution (0.1% BSA in PBS) 48 hrs after injection. The animals (n=3 for each time point) were sacrificed 6 hrs after pump implantation to examine the relative time-dependent expression of the genes possibly related to kainic acid-induced neurogenesis. Brains were immediately removed and hippocampi were sampled. The tissue was stored under -80C until use.

*Real-time RT-PCR*. Frozen tissue was homogenized in a glass homogenizer with an RNA extraction buffer (RNeasy Mini kit, QIAGEN). The homogenate was passed through QIAshredder (QIAGEN) and subjected to total RNA extraction following manufacturer’s instruction. The quantity and OD 260/280 of extracted total RNA was determined by spectrophotometry and only sample showed >1.8 was used for reverse transcription. 1 or 2 ug of total RNA was treated with DNaseI (Invitrogen) and subsequently converted to cDNA using SuperScript III First-Strand Synthesis System for RT-PCR (Invitrogen) according to manufacturer’s instruction. cDNA was stored under -20C until use. The expression level of each gene was examined by real-time RT-PCR using SYBR green. 2.5uL of appropriately diluted cDNA was added to PCR reaction mixture containing QuantiTect SYBR green PCR master mix (QIAGEN) and 0.3uM of each gene-specific primer. The primers were designed in Primer3 (<http://frodo.wi.mit.edu/>) and their specificity was checked by electrophoresis following PCR reaction. The primers used for each gene are listed in Table (Supplemental Table 1). Each sample was analyzed in at least duplicate. Real-time RT-PCR reaction was performed in RotorGene 3000 (Corbett Life Science) or ABI7500 (Applied Biosystems) with a following thermal cycle: initial 15min at 95°C followed by 40 cycles of 15sec at 94°C, 15sec at 60°C and 30sec at 72°C. Melting point analysis and/or electrophoresis was performed to check the reaction specificity. The expression level of each gene was determined by comparing it to the known standards using the software provided with each real-time PCR system. The relative expression level was determined by normalizing the expression level of each gene with that of GAPDH and expressed in ratio with the expression level in the naïve animal set as 1.0.

*Statistical analysis*. The experimental data are expressed as mean±SEM. The change in relative expression level was tested for statistical significance using one-way ANOVA followed by *post hoc* multiple comparison Tukey-Kramer test. The change with at least p <0.05 compared with both PBS and naïve counterparts was considered to be significant.
