# Supplementary material for: Activin A Is Essential for Neurogenesis Following Neurodegeneration
Source: Stem Cells. 2009 Jun;27(6):1330–46. doi: 10.1002/stem.80 (PMC2733378; doi:10.1002/stem.80)
Supplement: Supplementary file 4 [file stem0027-1330-SD6.doc]

Table II. Sequence of primers used in the real-time RT-PCR

Gene	Access No.	Type	Sequence	Amplicon(bp)
Gapdh	NM_001001303	Forward	AACTTTGGCATTGTGGAAGGG	273
		Reverse	TCATCATACTTGGCAGGTTTCTCC	
Inhibin α	NM_010564	Forward	GGAAGATGTCTCCCAGGCTA	124
		Reverse	GGATGGCCGGAATACATAAG	
Activin βA	NM_008380	Forward	CCTCGGAGATCATCACCTTT	118
		Reverse	AGGAAGAGCCACACTTCTGC	
Activin βB	NM_008381	Forward	AGAGCTTTTGGTTGCTTTGC	180
		Reverse	GCCACTTCTGGACACACGTA	
Activin βC	AK131946	Forward	GCCTACCACAAGCAATGTCA	90
		Reverse	GGTCAGGGAGGCAGAGTAGA	
Activin βE	NM_008382	Forward	CAGAAGCGAAGTGTGGTTTG	190
		Reverse	GTCTTGAACTCACGGTGCTG	
Bmp1	NM_009755	Forward	TTTGATGGCTACGACAGCAC	82
		Reverse	CTCCAGCCGAGTACACTTCC	
(Continued)				
Bmp2	NM_007552	Forward	AGATCTGTACCGCAGGCACT	142
		Reverse	[illegible]	
Gdf5	NM_008109	Forward	[illegible]	138
		Reverse	ATCCTTCTCCAAGGCACTGA	
Bmp3	NM_173404	Forward	GAACTCACAGATGCTCCATCC	134
		Reverse	[illegible]	
Gdf6	NM_013526	Forward	[illegible]	91
		Reverse	ATTAGCATTCCGACCGTTTC	
Bmp4	NM_007554	Forward	GATCTTTACCGGCTCCAGTCT	130
		Reverse	[illegible]	
Gdf7	NM_013527	Forward	[illegible]	147
		Reverse	TCGCACTGTCCCTGTCTG	
Bmp5	NM_007555	Forward	AACAAGCCTGCAAGAAGCA	193
		Reverse	[illegible]	
Gdf8	NM_010834	Forward	[illegible]	104
		Reverse	GCAGTCAAGCCCAAAGTCTC	
Bmp6	NM_007556	Forward	AAGGCTACGCTGCCAACTAC	113
		Reverse	[illegible]	
Gdf9	NM_008110	Forward	[illegible]	170
		Reverse	TAGCAAGACCGATTTGAGCA	
Bmp7	NM_007557	Forward	CTGGATCATTGCACCTGAAG	140
		Reverse	[illegible]	
Gdf10	NM_145741	Forward	[illegible]	109
		Reverse	AGCTCTGACGATGCTCTGG	
Bmp8a	NM_007558	Forward	ACCATTCATTGTGGCAGTTG	128
		Reverse	[illegible]	
Gdf11	AF092734	Forward	[illegible]	84
		Reverse	GAGTGTTCATCGCAGTCCAG	
Bmp8b	NM_007559	Forward	CACCTTGACAACAGCCAGAA	109
		Reverse	[illegible]	
Tgfα	NM_031199	Forward	[illegible]	104
		Reverse	GCTGGGCTTCTCATGTCTG	
Bmp10	NM_009756	Forward	TGCCGTCTGCTAACATCATC	187
		Reverse	[illegible]	
Tgfβ1	NM_011577	Forward	[illegible]	102
		Reverse	CGGGTTGTGTTGGTTGTAGA	
Bmp15	NM_009757	Forward	AAAGAAGCCCAGCATGTCTC	86
		Reverse	[illegible]	
Tgfβ2	NM_009367	Forward	[illegible]	100
		Reverse	TGGTGTTGTACAGGCTGAGG	
Gdf1	NM_008107	Forward	ATGCCACGTGGAGGAACTA	139
		Reverse	[illegible]	
Tgfβ3	NM_009368	Forward	[illegible]	122
		Reverse	TCTGGGTTCAGGGTGTTGTAT	
Gdf2	NM_019506	Forward	GGACTCCACAGAGGACAAGG	178
		Reverse	[illegible]	
Egf	NM_010113	Forward	[illegible]	165
		Reverse	GGAGGGTAGGTGGGAGTTTC	
Fgf2	NM_008006	Forward	GGCTGCTGGCTTCTAAGTGT	119
		Reverse	TCGTTTCAGTGCCACATACC	
ActrI	NM_007394	Forward	CAACAGAGGCCAAACATACCT	104
		Reverse	AGTCTTGCGGATGGGTTCT	
ActrIIb	NM_007397	Forward	AGATGAGGCCCACGATTAAG	137
		Reverse	CAGGGATACCCGCTCTTCTA	
